# Wnt antagonism without TGFβ induces rapid MSC chondrogenesis via increasing AJ interactions and restricting lineage commitment

**DOI:** 10.1016/j.isci.2022.105713

**Published:** 2022-12-02

**Authors:** Chen-Chan Hsieh, B. Linju Yen, Chia-Chi Chang, Pei-Ju Hsu, Yu-Wei Lee, Men-Luh Yen, Shaw-Fang Yet, Linyi Chen

**Affiliations:** 1 Institute of Molecular Medicine, National Tsing Hua University, Hsinchu, Taiwan; 2 Regenerative Medicine Research Group, Institute of Cellular and System Medicine, National Health Research Institutes (NHRI), 35 Keyan Road, Zhunan, Miaoli County35053, Taiwan; 3 Graduate Institute of Life Sciences, National Defense Medical Center (NDMC), Taipei, Taiwan; 4 Department of Obstetrics/Gynecology, National Taiwan University (NTU) Hospital and College of Medicine, NTU, Taipei, Taiwan

**Keywords:** Bioengineering, Biological sciences, Molecular medicine, Tissue engineering

## Abstract

Human mesenchymal stem cells (MSCs) remain one of the best cell sources for cartilage, a tissue without regenerative capacity. However, MSC chondrogenesis is commonly induced through TGFβ, a pleomorphic growth factor without specificity for this lineage. Using tissue- and induced pluripotent stem cell-derived MSCs, we demonstrate an efficient and precise approach to induce chondrogenesis through Wnt/β-catenin antagonism alone without TGFβ. Compared to TGFβ, Wnt/β-catenin antagonism more rapidly induced MSC chondrogenesis without eliciting off-target lineage specification toward smooth muscle or hypertrophy; this was mediated through increasing N-cadherin levels and β-catenin interactions—key components of the adherens junctions (AJ)—and increasing cytoskeleton-mediated condensation. Validation with transcriptomic analysis of human chondrocytes compared to MSCs and osteoblasts showed significant downregulation of Wnt/β-catenin and TGFβ signaling along with upregulation of α-catenin as an upstream regulator. Our findings underscore the importance of understanding developmental pathways and structural modifications in achieving efficient MSC chondrogenesis for translational application.

## Highlights

• Classical TGFβ protocol for MSC chondrogenesis results in off-target differentiation
• More rapid chondrogenesis is seen using Wnt antagonism only without TGFβ
• Wnt antagonism only without TGFβ increase N-cadherin/β-catenin AJ interactions
• Wnt & TGFβ pathways decreased & AJ upregulated in human chondrocyte transcriptome

## Graphical abstract

**Figure:**
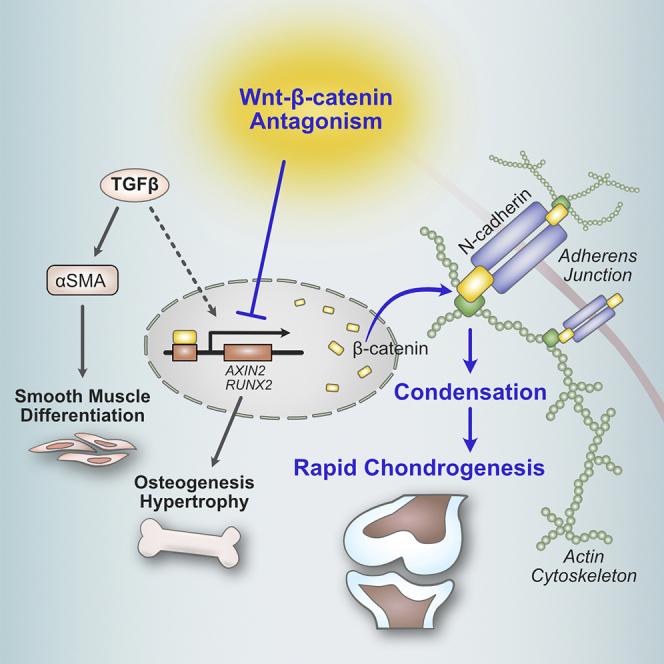

## Introduction

Cartilage and joint diseases are one of the most common clinical conditions affecting quality of life worldwide because of increases in the two major risk factors of age and obesity. Current treatments for arthritic diseases are largely limited to symptom palliation using analgesics for mild cases, and immunosuppressants and/or surgery for more serious cases.^1^ However, these treatments are not disease-modifying or curative; moreover, cartilage is unable to regenerate. Although more novel treatment using autologous cartilage tissue or chondrocytes are being tested, the rarity of the cell/tissue source, *ex vivo* culture difficulties, and low cell viability after transplantation have resulted in limited success with these methods.^2^ Thus, continued investigation into more efficacious methods of regenerating cartilage is ongoing to find curative treatments for these common and debilitating diseases.

Human multipotent mesenchymal stem cells (MSCs) are versatile somatic stem cells with immunomodulatory properties. First isolated in adult bone marrow (BM), MSCs have subsequently been found in numerous post-natal organ/tissues^3^ as well as directly differentiated from pluripotent stem cells such as human embryonic stem cells (ESCs)^4^^,^^5^ and induced pluripotent stem cells (iPSCs).^6^^,^^7^ MSCs readily differentiate into chondrocytes, osteoblasts, adipocytes, and fibroblasts, and their easy accessibility compared to many other cell types including chondrocytes render them ideal for use in cartilage-related diseases. However, chondrogenic differentiation efficiency is low because of the requisite cumbersome 3-dimensional (3D) pellet culture and the relative lack of knowledge on molecular mechanisms involved in chondrogenesis, compared to osteogenesis and adipogenesis. The most common factors used to induce MSC chondrogenesis are TGFβ1 and TGFβ3, two pleomorphic growth factors, but information on mechanisms involved for either of these factors are surprisingly scarce despite decades of use.^8^ Moreover, TGFβ is well known to induce fibrosis, as well as ossification in cartilage.^9^^,^^10^ Thus, there clearly is a need for more precision and efficiency in achieving MSC chondrogenesis for therapeutic application.

β-catenin/Wnt signaling, a major developmental and oncogenic pathway, is also important in skeletal related-tissue/organ development, especially for bone and cartilage tissue. Clinical studies have found that the low-density lipoprotein receptor-related protein 5 (LRP5), a key component of canonical Wnt/β-catenin pathway, is critical to osteogenesis, with a loss-of-function mutation impeding bone development to cause osteoporosis-pseudoglioma syndrome^11^ and a gain-of-function mutation increasing bone mass.^12^ In addition, transgenic mouse studies have demonstrated that impairment of Wnt signaling enhances cartilage development but impedes bone formation,^13^ and cell-based studies show that Runt-related transcription factor 2 (RUNX2), the master transcription factor for osteogenesis and also involved in ossification of hypertrophic cartilage, is a downstream gene of β-catenin transcriptional activity.^14^ Critically, β-catenin serves not only as a transcription factor but also as a key structural component of the adherens junctions (AJs), an important complex mediating cell-cell adhesion and cytoskeletal changes^15^—activities which are important during chondrogenesis.^16^^,^^17^ We therefore hypothesized that inhibition of the Wnt/β-catenin pathway may more precisely induce MSC chondrogenesis than TGFβ. To ascertain this, we utilized multiple sources of human MSCs—including iPSC-derived MSCs (iPSC-MSCs), ESC-derived MSCs (ESC-MSCs), and BM-MSCs—and also explored the role of AJ-β-catenin interactions in MSC chondrogenesis.

## Results

### Wnt/β-catenin antagonism significantly enhanced MSC chondrogenesis whereas agonism resulted in the opposite and upregulation of the master osteogenic transcription factor Runx2

**Figure 1:**
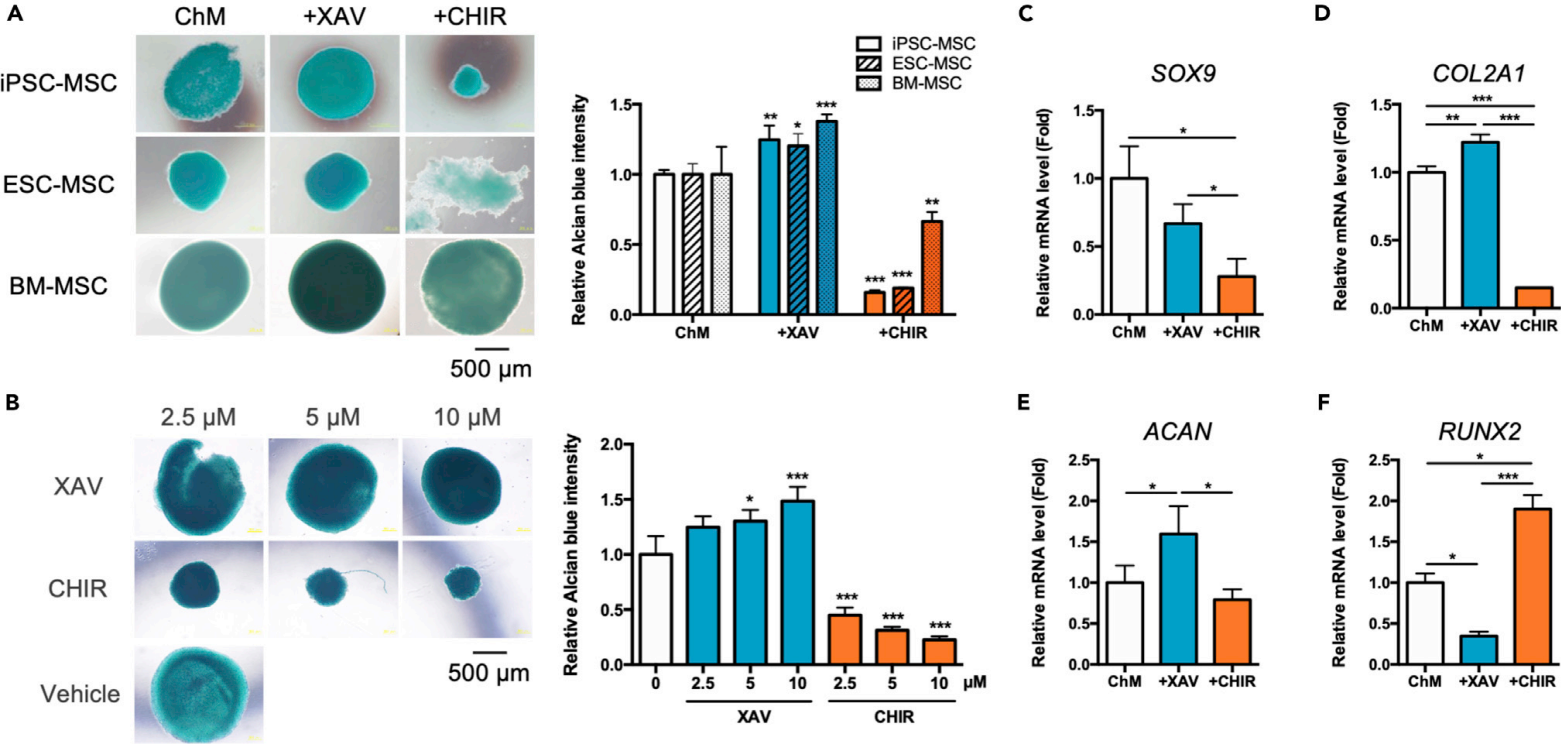
Wnt/β-catenin antagonism enhances human mesenchymal stem cell (MSC) chondrogenesis while agonism suppress chondrogenesis and upregulate master osteogenic transcription factor RUNX2 (A) Alcian blue staining (left panel) of 3D pellet-cultured human induced pluripotent stem cell-derived MSCs (iPSC-MSCs), embryonic stem cell-derived MSCs (ESC-MSCs), and bone marrow-MSCs (BMMSCs) treated with 10 μM of either the Wnt/β-catenin antagonist XAV939 (XAV) or the agonist CHIR99021 (CHIR) in complete chondrogenic medium containing TGFβ3 (ChM) for 20 days. Quantification of Alcian blue staining (right graph) was performed, with comparisons of Wnt antagonism or agonism to ChM for each MSC type. Scale bar, 500 μm. (B) Alcian blue staining (left panel) and quantification (right panel) of pellet-cultured iPSC-MSCs treated with either XAV or CHIR at the indicated concentrations in ChM for 20 days. Scale bar, 500 μm. (C–F) Gene expression levels of the chondrogenic genes (C) *SOX9*, (D) collagen 2A1 (*COL2A1*), and (E) aggrecan (*ACAN*), as well as (F) *RUNX2*, the osteogenic master transcription factor, in 3D pellet-cultured iPSC-MSCs treated with either XAV or CHIR in ChM cultured for 3 days as analyzed by qPCR. Data are represented as mean ± SD One-way ANOVA: ∗, p< 0.05, ∗∗, p< 0.01, ∗∗∗, p< 0.001.

To investigate the effects of Wnt modulation on MSC chondrogenesis, we treated 3D pellet-cultured human MSCs—including iPSC-MSCs, ESC-MSCs and BM-MSCs— in a standard complete chondrogenic induction medium (ChM) containing TGFβ3 along with a small molecule Wnt/β-catenin agonist CHIR which inhibit GSK3β to disrupt the β-catenin destruction complex, or antagonist XAV, a tankyrase inhibitor which stabilizes the β-catenin destruction complex. TGFβ3 was utilized in ChM because it has been found to possess higher chondrogenic potential than TGFβ1.^18^ We found that in ChM conditions, Wnt inhibition by XAV increased condensation of the pellets whereas agonism by CHIR decreased pellet integrity in all 3 sources of MSCs at Day 20. Quantification of glycosaminoglycans (GAGs) showed a significant increase in the expression of this structural extracellular matrix of cartilage with Wnt/β-catenin antagonism, whereas the opposite was seen with Wnt/β-catenin agonism (Figure 1A). Because iPSC-MSCs can be patient-specific as well as continually derived, we focused on using this source in further studies. By observing morphological characteristics, including pellet size and integrity, and quantification of GAGs (Figure 1B), we found a dose-dependent effect of Wnt/β-catenin modulation on MSC chondrogenesis. Analysis of chondrogenic gene expression levels after Wnt/β-catenin antagonism at Day 3 showed significant upregulation of two key chondrogenic genes collagen 2A1 (*COL2A1*) and aggrecan (*ACAN*) but not *SOX9*; Wnt/β-catenin agonism, on the other hand, resulted in significant downregulation of all 3 genes (Figures 1C–1E). Of interest, Wnt/β-catenin antagonism decreased expression of the master osteogenic transcription factor *RUNX2* whereas agonism showed the opposite effect (Figure 1F). These findings collectively demonstrate that Wnt antagonism significantly enhances MSC chondrogenic differentiation whereas agonism results in the opposite effect and upregulates the master osteogenic transcription factor *RUNX2*.

### TGFβ rapidly increased alpha smooth muscle actin (*α*SMA) and RUNX2 expression in MSCs during chondrogenic induction

**Figure 2:**
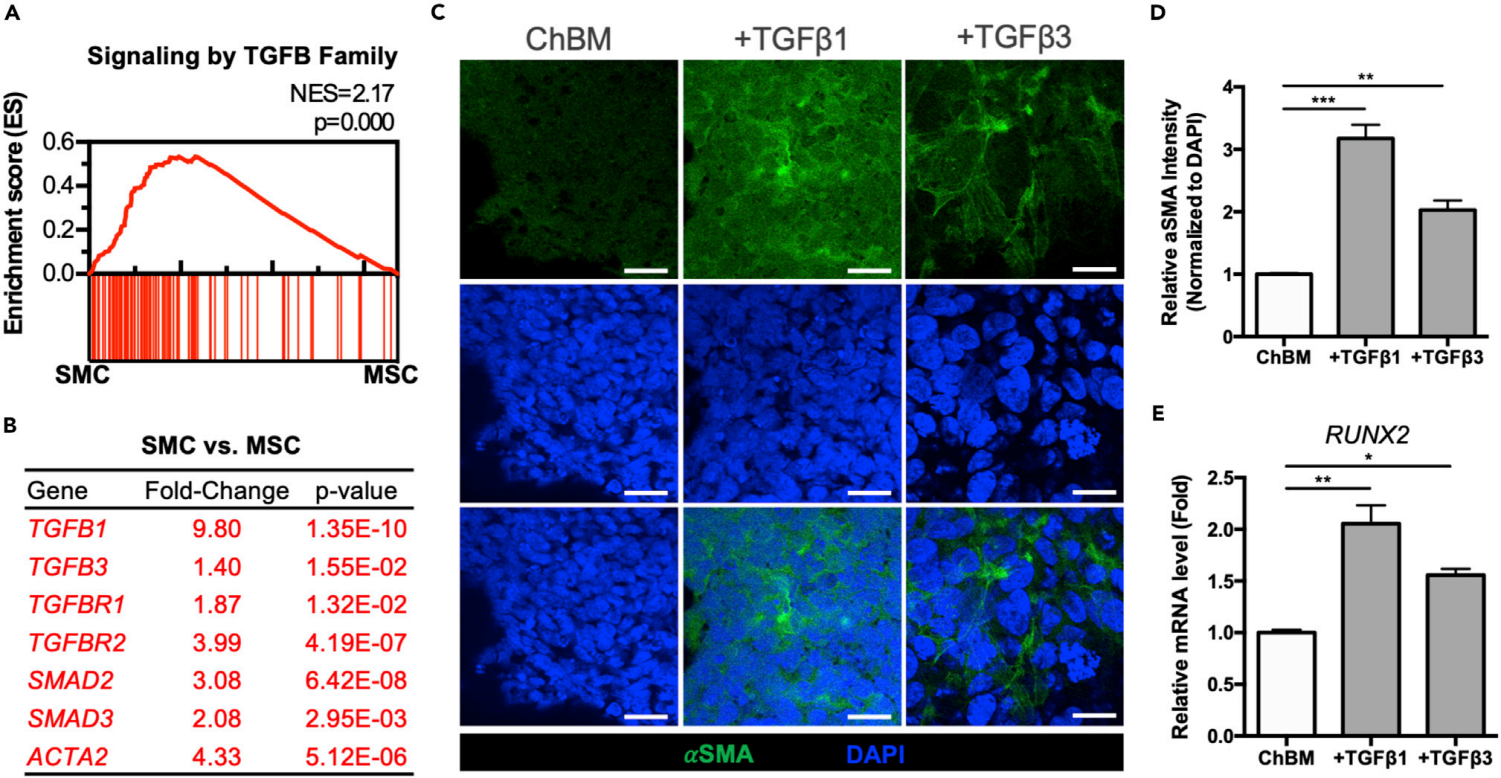
Gene set of signaling by TGFβ family are enriched in human smooth muscle cells compared to MSCs, and TGFβ rapidly increases *α*-smooth muscle actin (*α*SMA) and RUNX2 expression in MSCs during chondrogenic induction (A) GSEA enrichment plot of signaling by TGFβ family members in human BM-MSCs (MSC; GSE128949) compared to smooth muscle cells (SMC; GSE109859). The normalized enrichment score (NES) and nominal p values are shown. (B) Relative changes of mean expression levels of selected relevant genes and the p value in human primary SMCs compared to BM-MSCs (SMC versus MSC). Genes with significant upregulation (p < 0.05, fold-change>1) are colored in red. (C and D) Confocal immunofluorescence detection (scale bar, 20 μm) and (D) quantification of *α*SMA on Day 3 of pellet-cultured iPSC-MSCs in ChBM alone or with addition of TGFβ1 or TGFβ3; nuclei are labeled by 4′,6-diamidino-2-phenylindole (DAPI) and *α*SMA fluorescent intensity was normalized to nuclear staining (DAPI). (E) Gene expression levels of *RUNX2* as quantified by qPCR on Day 3 of pellet-cultured iPSC-MSCs in ChBM alone or with addition of TGFβ1 or TGFβ3. Data are represented as mean ± SD. One-way ANOVA: ∗, p< 0.05, ∗∗, p< 0.01, ∗∗∗, p< 0.001.

Although TGFβ1 and TGFβ3 have long been used for MSC chondrogenesis, it is also well established that TGFβ can induce smooth muscle differentiation^9^ and upregulate an osteogenic program.^19^ By analyzing the transcriptomic data of human primary BM-MSCs and smooth muscle cells from public database (GSE128949 for human primary BM-MSCs; GSE109859 for human primary smooth muscle cells) with Ranked Gene Set Enrichment Analysis (GSEA), gene set of Signaling by TGFβ Family Members was positively enriched in human BM-MSCs compared to smooth muscle cells with normalized enrichment score (NES) equals to 2.17 and the nominal p value less than 0.001 (Figure 2A). Relative changes in mean expression levels also showed that the core components in TGFβ signaling (*TGFB1*, *TGFB3 TGFBR1*, *TGFBR2*, *SAD2* and *SMAD3*) as well as *α*SMA (*ACTA2*), a marker of smooth muscle differentiation and also fibrosis,^20^ were all significantly upregulated in human primary smooth muscle cells (SMC) compared to MSC (Figure 2B). These results indicated that TGFβ signaling is positively correlated with the smooth muscle lineage. In addition to smooth muscle lineage, TGFβ/BMP signaling is also known to induce MSC osteogenic differentiation.^21^ To further address the potential off-target effects of TGFβ in MSC chondrogenic differentiation, we assessed whether TGFβ upregulates expression of *α*SMA and/or *RUNX2*, the master osteogenic transcription factor, during MSC chondrogenesis. Immunofluorescent staining of *α*SMA on Day 3 of MSC chondrogenic induction showed strong expression in both TGFβ-treated groups compared to chondrogenic basal medium (ChBM) which does not contain TGFβ (Figure 2C), with quantitative fluorescent intensity demonstrating significantly higher *α*SMA expression (up to 3-fold) in TGFβ-treated groups compared to ChBM (Figure 2D). *RUNX2* mRNA expression was also significantly upregulated in TGFβ1-and TGFβ3-treated groups on Day 3 compared to ChBM (Figure 2E). These results indicate that TGFβ rapidly induce non-chondrogenic lineages including smooth muscle and ossification during MSC chondrogenesis.

### Wnt/β-catenin antagonism alone induced more rapid MSC chondrogenesis than TGFβ

**Figure 3:**
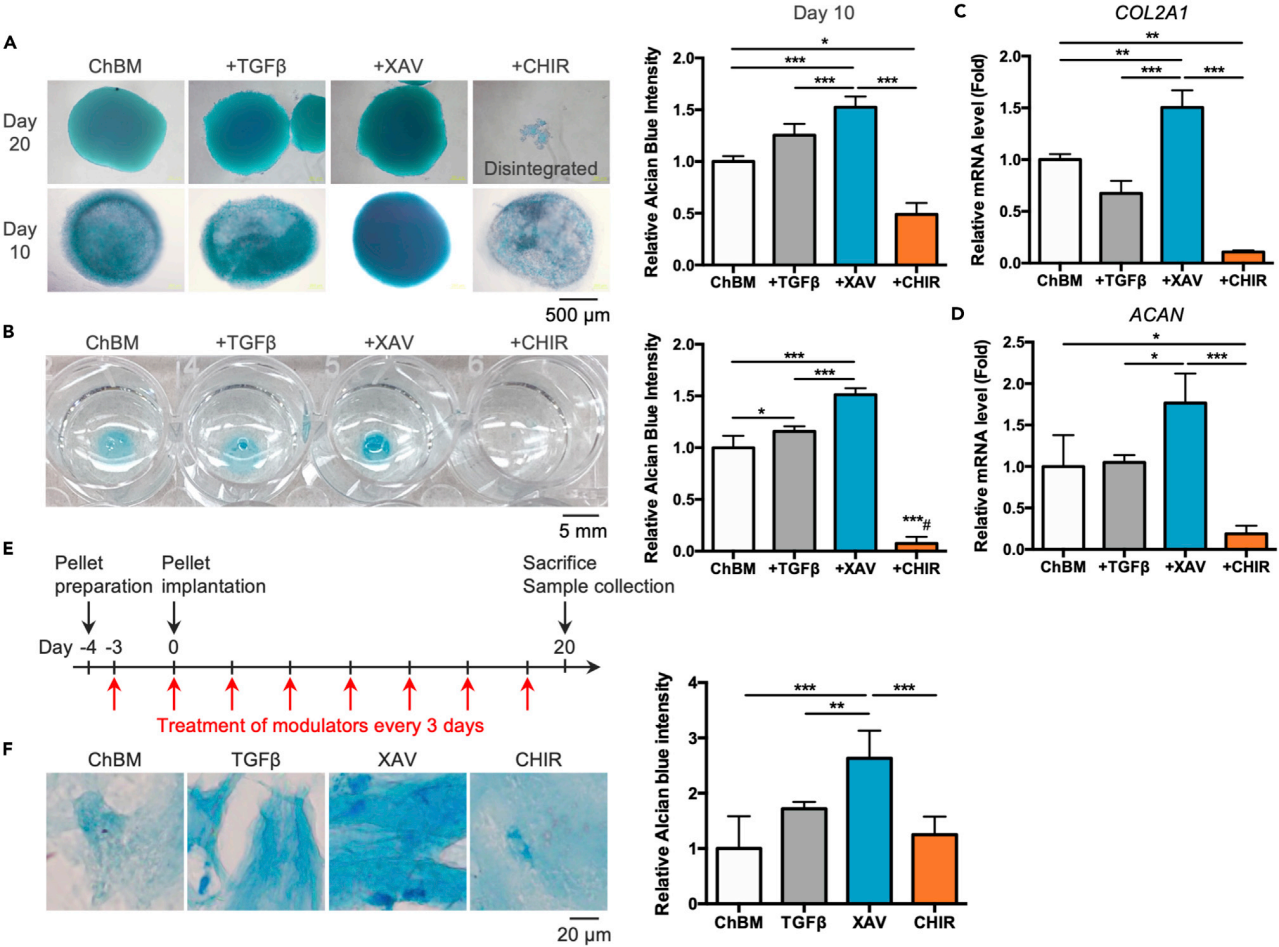
Wnt/β-catenin antagonism alone induced more rapid MSC chondrogenesis than TGFβ *in vitro* and *in vivo* (A) Alcian blue staining of pellet-cultured iPSC-MSCs treated with the indicated modulators (10 ng/mL TGFβ3, 10 μM CHIR, or 10 μM XAV) at Day 20 and Day 10 (left top and bottom panels, respectively), with absorbance quantification for Day 10 results (right panel). Scale bar, 500 μm. (B) Alcian blue staining (left panel) and absorbance quantification (right panel) of micromass-cultured iPSC-MSCs treated with the indicated modulators for 10 days. Scale bar, 5 mm. (C and D) Expression levels of chondrogenic gene (C) *COL2A1* and (D) *ACAN* in 3-day pellet-cultured MSCs treated with the indicated modulators for 3 days as quantified by qPCR. (E) Schematic procedure of *in vivo* experimentation. Mouse BM-MSCs were cultured as 3D pellets in ChBM first then transplanted subcutaneously into wildtype mouse. Modulators (10 μM of CHIR, XAV, or 10 ng/mL of TGFβ3) were injected locally every 3 days until harvest at Day 20. (F) Alcian blue staining (left panel) and absorbance quantification (right panel) of harvested tissue sections from transplanted MSCs treated with the indicated modulators at Day 20. Scale bar, 500 μm. Data are represented as mean ± SD One-way ANOVA: ∗, p < 0.05, ∗∗, p < 0.01, ∗∗∗, p < 0.001.

Because Wnt/β-catenin antagonism strongly enhances MSC chondrogenesis and TGFβ agonism significantly induce other non-chondrogenic lineage markers during this process, we examined the possibility of replacing TGFβ completely with Wnt/β-catenin antagonism to achieve more specific MSC chondrogenic differentiation. After 20 days of chondrogenic differentiation, MSC pellets treated with either TGFβ3 or XAV formed well-condensed spheres and strong Alcian blue staining compared to ChBM, whereas pellets treated with CHIR did not undergo condensation but became disintegrated at the end of the culture time period (Figure 3A, top panel). Strikingly, we found that GAG production as evidenced by Alcian blue staining was more rapidly induced in pellets cultured with XAV than TGFβ3 at Day 10, approximately halfway through the full differentiation time period (Figure 3A, bottom panel). This was verified with quantification of GAG production at Day 10 in which significant increases in MSC pellets cultured with XAV was seen, not only when compared to ChBM culture but also to TGFβ3 treatment, whereas CHIR treatment resulted in minimal GAG production (Figure 3A, right panel). Moreover, induction of chondrogenesis by Wnt antagonism at this earlier time point was not further enhanced with TGFβ3 supplementation, implicating that there is no synergistic effect (Figure S1). Histological cross-sections of Day 10 pellets also revealed stronger Alcian blue staining representing GAG production, as well as a more solid and homogeneous pellet structure in XAV-treated conditions, compared to TGFβ-treated or ChBM cultured conditions (Figure S2). Immunohistochemical staining for chondrogenic markers type II collagen (COL2) and aggrecan (ACAN) also showed stronger signals in XAV-treated conditions compared to TGFβ- or ChBM-conditions (Figure S3). We further validated these findings using micromass culture, which is a more convenient method to induce chondrogenic differentiation in standard 2D culture condition and useful for *in vitro* high-throughput drug screening.^22^ Similar to 3D pellet culture, micromass culture of MSCs with XAV treatment at Day 10 demonstrated better structural condensation and stronger Alcian blue intensity compared to ChBM culture and even TGFβ3-treated culture, whereas CHIR treatment resulted in minimal evidence of any micromass structure or Alcian blue staining. Quantitation of GAG production was in line with the morphologic findings, with XAV treatment resulting in the highest production of GAG (Figure 3B). XAV treatment also most significantly upregulated expression of both chondrogenic genes *COL2A1* and *ACAN*, whereas TGFβ3 treatment did not increase expression of either genes and CHIR treatment resulted in minimal expression of either genes at Day 3 (Figures 3C and 3D). To further ascertain that the outcome is not specific to the utilized antagonist and/or agonist, iCRT-3, a non-tankyrase Wnt antagonist^23^ and lithium chloride (LiCl) a well-studied Wnt/β-catenin pathway agonist^24^ were additionally used (Figure S4). At Day 3 and Day 5, pellets cultured with either XAV or iCRT-3 resulted in condensed and spherical morphology while progressive disintegration was seen of pellets cultured with either CHIR or LiCl (Figure S4A). Moreover, Alcian blue staining and quantification at the early time point of Day 6 showed that both XAV and iCRT-3 already induced significant increases in GAG production not only over baseline/control levels but also at a level similar to or better than with TGFβ3 (Figure S4B). These results strongly indicate that antagonism of the Wnt/β-catenin pathway rather than antagonist-specific effects is responsible for the rapid induction of MSC chondrogenesis.

To validate our *in vitro* data, we performed subcutaneous transplantation into wild-type mice of mouse BM-MSCs cultured in ChBM medium with injections of various modulators alone (TGFβ3, XAV, or CHIR) every three days for 20 days (Figure 3E). Histological analyses of *in vivo* differentiated samples demonstrated that the highest level of GAG production was in the XAV-treated group; moreover, Wnt/β-catenin antagonism significantly increased GAG production compared to TGFβ3 agonism (Figure 3F). These results indicated that Wnt inhibition significantly enhances MSC chondrogenesis *in vivo*. All these results demonstrate that replacement of TGFβ with Wnt/β-catenin antagonism achieves more rapid and specific MSC chondrogenesis. We further performed gene expression array of two iPSC-MSCs and one bone marrow-derived MSC with XAV- or TGFβ-based chondrogenic induction. GSEA results revealed that chondrogenic pathways are positively enriched in both conditions, whereas gene sets with regards to bone development and smooth muscle differentiation are negatively enriched in XAV- compared to TGFβ-induced chondrogenesis; moreover, Wnt antagonism does not enrich for adipogenesis, demonstrating the strong lineage-specificity of inhibiting this pathway for chondrogenesis. As for adipo-related lineage, we found no significant difference in the enrichment of Adipose tissue development gene set, indicating that Wnt antagonism is indeed highly specific for MSC chondrogenesis (Figure S5). Collectively, these results demonstrate that compared to TGFβ, MSC chondrogenesis using Wnt antagonism is highly specific and not result in off-target lineage commitment toward smooth muscle lineage or hypertrophy.

### Wnt/β-catenin antagonism but not TGFβ agonism during MSC chondrogenesis decreased canonical Wnt/β-catenin transcriptional activity including RUNX2 expression

**Figure 4:**
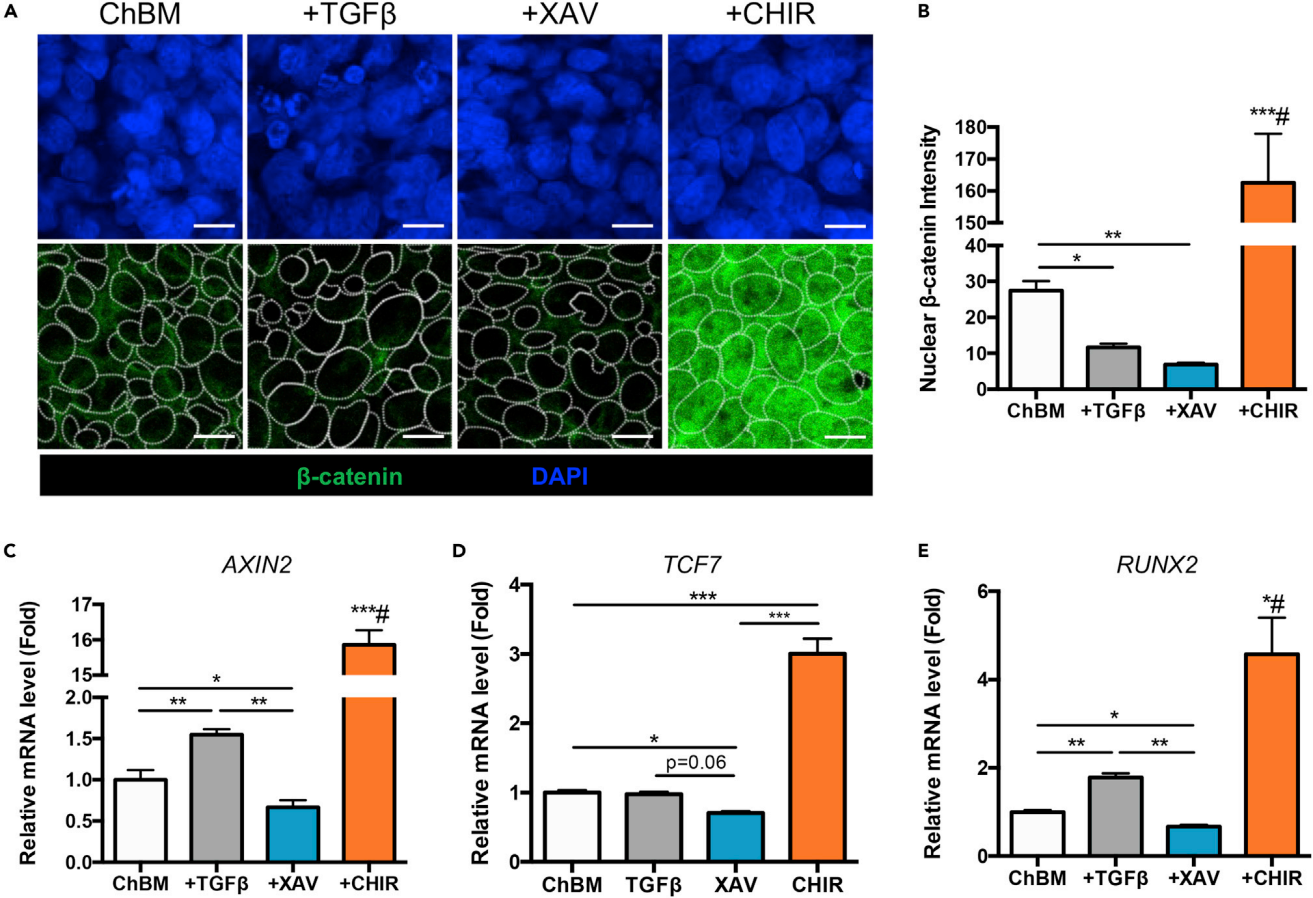
Wnt/β-catenin antagonism but not TGFβ agonism during MSC chondrogenesis decreased canonical Wnt/β-catenin transcriptional activity including RUNX2 expression (A) Immunofluorescent staining of β-catenin in iPSC-MSC pellets cultured in ChBM only or with addition of TGFβ3 (10 ng/mL), CHIR (10 μM), or XAV (10 μM) at 1 day. Dotted line indicates nuclear borders (stained with DAPI). Scale bar, 10 μm. (B) Quantification of nuclear β-catenin intensity. (C and D) Gene expression levels of (C) *AXIN2,* (D) *TCF7* and (E) *RUNX2* in iPSC-MSC pellets cultured in ChBM only or with addition of TGFβ3 (10 ng/mL), CHIR (10 μM), or XAV (10 μM) for 3 days as quantified by qPCR. #, compared to all other groups. Data are represented as mean ± SD One-way ANOVA: ∗, p< 0.05, ∗∗, p< 0.01, ∗∗∗, p< 0.001.

The significant enhancement of MSC chondrogenesis with Wnt/β-catenin antagonism alone led us to investigate the role of the canonical Wnt/β-catenin pathway during the differentiation process. As a transcription factor, β-catenin undergoes translocation from the cytoplasm to the nucleus when activated. Using immunofluorescent staining and quantification, we found that pellet-cultured MSCs treated with XAV had the lowest nuclear β-catenin levels compared to control ChBM culture or TGFβ3 treatment, whereas CHIR treatment dramatically increased nuclear β-catenin levels (Figures 4A and 4B), which was expected because CHIR is known to strongly induce β-catenin transcriptional activity.^25^ Assessment of β-catenin pathway downstream gene expression levels demonstrated that after XAV treatment, expression of *AXIN2* and *TCF7*, two well-established β-catenin-activated downstream genes, and the osteogenic master transcription factor *RUNX2* were all significantly decreased, whereas CHIR treatment strongly increased expression of these genes as expected (Figures 4C and 4D). Surprisingly, TGFβ3 treatment resulted in increased expression of not only *RUNX2* but *AXIN2* as well, implicating a weak agonistic effect of this factor on canonical Wnt/β-catenin pathway. These findings demonstrate that inhibition of Wnt/β-catenin transcriptional activity is critical for MSC chondrogenesis, and that TGFβ treatment may not be optimal during this process because of its weak agonism for the pathway.

### Wnt/β-catenin antagonism but not TGFβ agonism increased N-cadherin expression and interactions with β-catenin at AJs as well as enhancing actin cytoskeleton-mediated condensation

**Figure 5:**
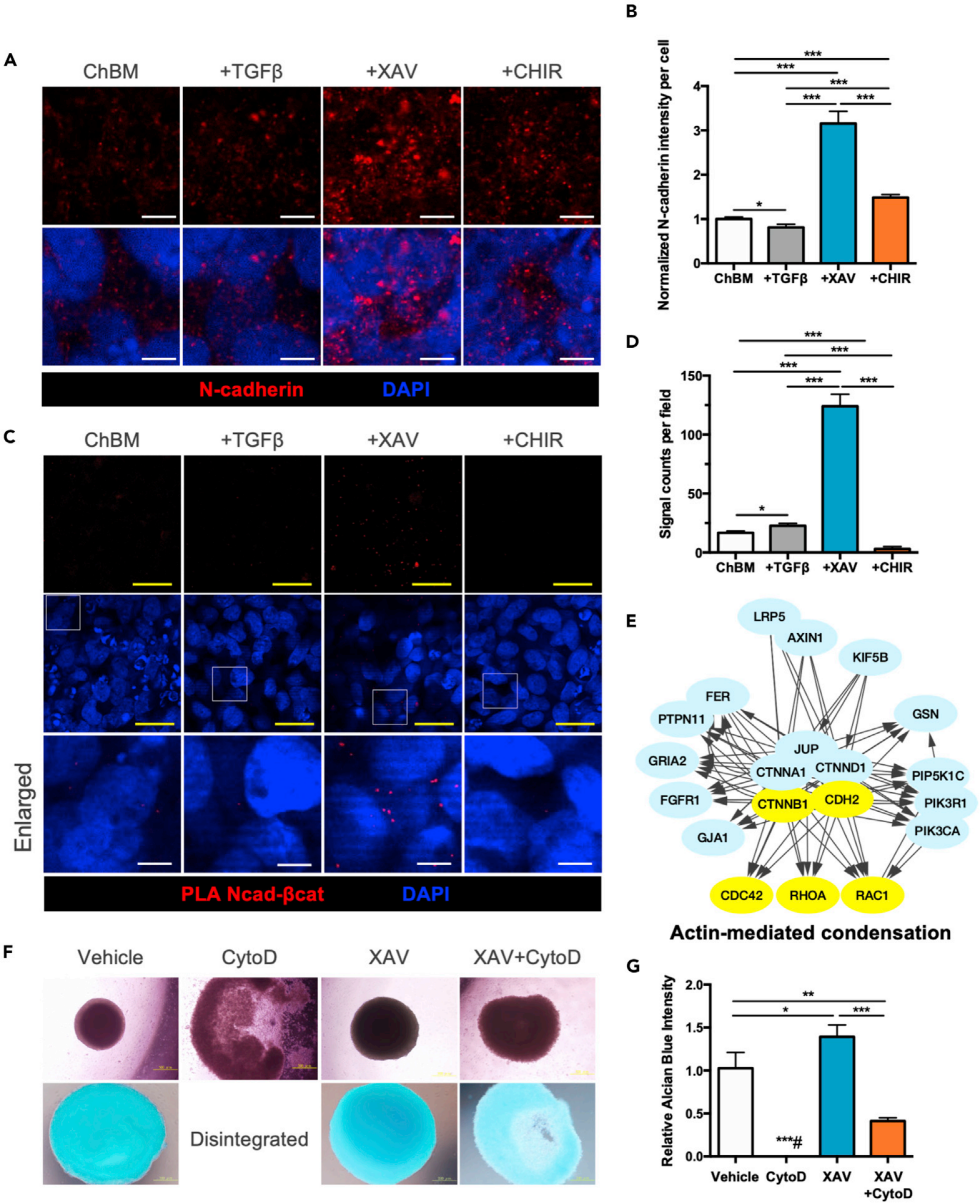
Wnt/β-catenin antagonism but not TGFβ agonism increases N-cadherin expression, and interactions with β-catenin as well as actin cytoskeleton-mediated condensation (A and B) Representative confocal immunofluorescence microscopy images and (B) quantification of N-cadherin expression in iPSC-MSCs cultured as micromass in ChBM only or with addition of TGFβ3 (10 ng/mL), CHIR (10 μM), or XAV (10 μM) for 1 day. Nuclei are labeled by DAPI. Scale bar, 5 μm. (C and D) Proximity ligation assay (PLA) for N-cadherin-β-catenin (Ncad-βcat) interaction and (D) quantification of signal counts per field of iPSC-MSCs cultured as micromass in the indicated chondrogenic conditions for 1 day. Nuclei are labeled by DAPI. Yellow scale bar, 20 μm. White scale bar, 5 μm. (E) Relationship of core elements in N-cadherin pathway derived from the Pathway Interaction Database. PID_NCADHERIN_PATHWAY filtered by first neighborhood of CTNNB1 and CDH2 are presented. (F and G) Images of phase contrast and Alcian blue staining images (top and bottom panel respectively) and (G) absorbance quantification of pellet-cultured iPSC-MSCs treated with the indicated modulators (0.25 μM cytochalasin D (CytoD), 10 μM XAV) at Day 10. Data are represented as mean ± SD One-way ANOVA: ∗, p< 0.05, ∗∗, p< 0.01, ∗∗∗, p< 0.001.

In addition to its role as a transcription factor, β-catenin is also a component of the AJ, a key structure of cell-cell adhesion which is a critical aspect during cartilage condensation.^26^ To investigate how Wnt/β-catenin modulation during MSC chondrogenesis affect AJs, we performed immunofluorescent staining for N-cadherin, a major AJ component in mesenchymal cell types like MSCs which is also essential during chondrogenesis.^27^ We found N-cadherin expression to be strongly and most significantly upregulated in MSC micromass culture with XAV treatment after 24 h, compared to all other conditions (Figures 5A and 5B). To further investigate whether there were increased interactions between β-catenin and the increased N-cadherin levels induced by Wnt/β-catenin antagonism, we performed proximity ligation assay (PLA) between these two molecules. We found that only XAV treatment during MSC chondrogenesis could significantly increase N-cadherin/β-catenin interactions as evidenced by increased PLA signals (Figures 5C and 5D). Of interest, whereas CHIR treatment resulted in the strongest expression of β-catenin (Figure S6), there was minimal PLA signal detected, indicating little interaction/colocalization between N-cadherin and β-catenin (Figures 5C and 5D). To validate the role of N-cadherin in Wnt antagonism-induced MSC chondrogenesis, N-cadherin blocking antibody was applied. Alcian blue staining at Day 6 showed that blocking of N-cadherin interactions significantly decreased the enhancement of chondrogenesis induced by XAV treatment (Figure S7). These findings demonstrate that Wnt inhibition enhances MSC chondrogenic differentiation through increasing N-cadherin levels and interaction/colocalization between N-cadherin and β-catenin at AJs.

To validate interactions between N-cadherin and β-catenin in MSC chondrogenic differentiation, we first examined the N-cadherin pathway using the Pathway Interaction Database (PID_NCADHERIN_PATHWAY) and found that with N-cadherin (CDH2), β-catenin (CTNNB1) is a core participant within the signaling pathway, with numerous components of the AJ: α-catenin (CTNNA1), p120/δ-catenin (CTNND1), and plakoglobin (JUP). Moreover, the three major GTPases responsible for actin cytoskeleton organization—RhoA, CDC42, and Rac1—are all found as downstream effectors in the signaling pathway (Figure 5E), demonstrating that actin cytoskeleton organization is highly regulated by the N-cadherin signaling pathway. To validate the bioinformatics analyses, during MSC chondrogenesis we treated with cytochalasin D (CytoD), an inhibitor of actin polymerization, which significantly disrupted the condensation process with the pellets completely disintegrated by Day 10. In contrast, cells treated with XAV+CytoD partially maintained the pellet structure and chondrogenic differentiation (Figures 5F and 5G). All these results suggest that Wnt/β-catenin antagonism promotes MSC chondrogenic differentiation by increasing N-cadherin expression and its interactions with β-catenin at AJs as well as enhancing actin cytoskeleton-mediated condensation.

### Significant downregulation of Wnt/β-catenin and TGFβ-related pathways in transcriptomes of human primary chondrocytes compared to osteoblasts

**Figure 6:**
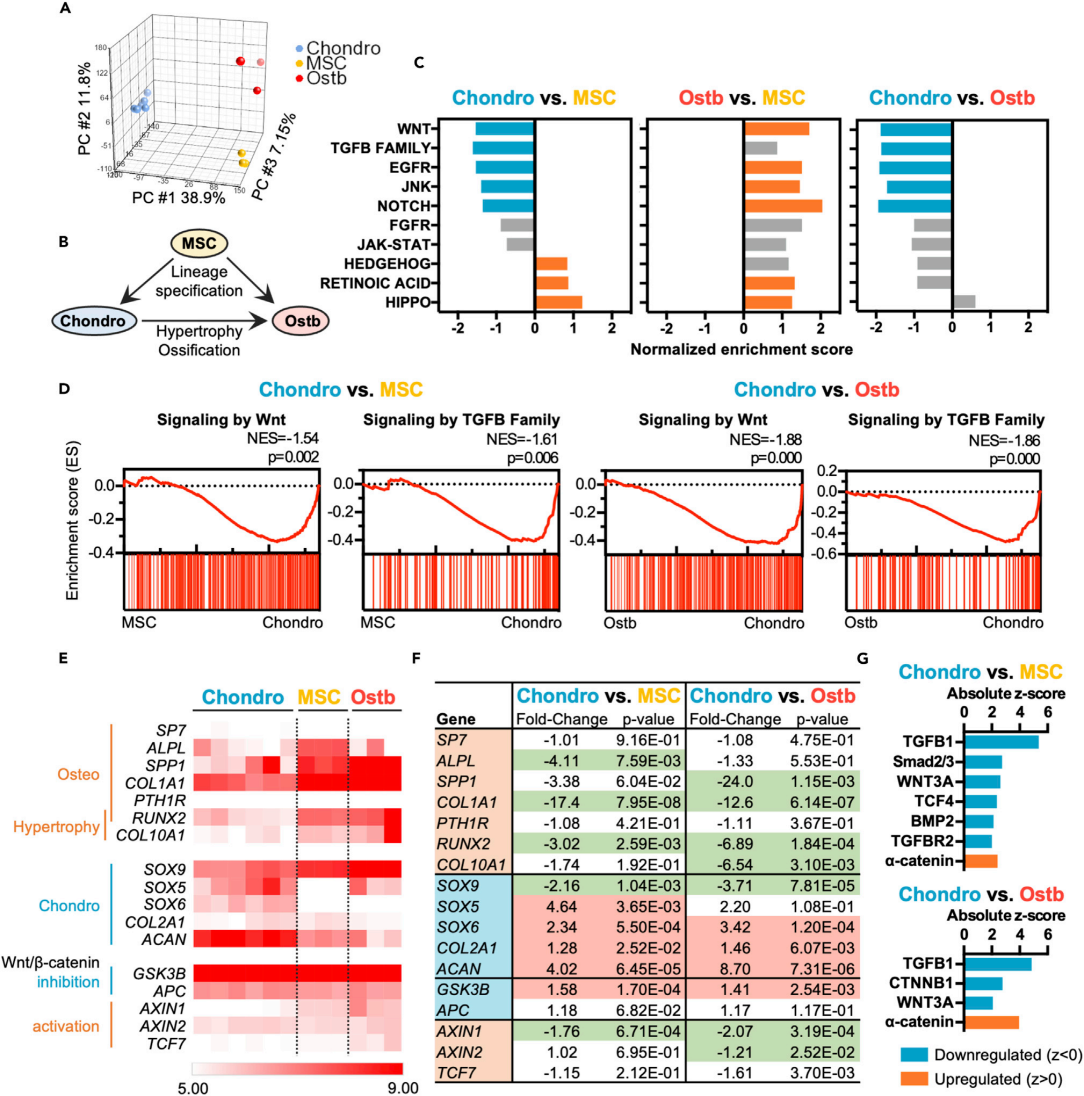
Wnt/β-catenin and TGFβ-related pathways are significantly downregulated in human primary chondrocytes compared to MSCs and osteoblasts (A and B) Principal component analysis (PCA) based on transcriptomic data and (B) interpreted relationship between human primary chondrocytes (Chondro), osteoblasts (Ostb) and BM-MSCs (MSC). (C) Enrichment analysis of major developmental pathways in human primary chondrocytes compared to MSCs (Chondro versus MSC), osteoblasts compared to MSCs (Ostb versus MSC) and chondrocytes compared to osteoblasts (Chondro versus Ostb) by Gene Set Enrichment Analysis (GSEA). Pathways in blue and orange are significantly enriched (p<0.05) with negative and positive normalized enrichment score respectively, non-significant pathways (p>0.05) were colored in gray. (D) GSEA enrichment plot of signaling by Wnt (stable Identifier: R-HSA-195721) and TGFβ family members (stable Identifier: R-HSA-9006936) in human primary chondrocytes compared to MSCs (left 2 panels) and osteoblasts (right 2 panels). The normalized enrichment score (NES) and nominal p values are shown. (E) Heatmap showing the Robust Multi-array Average (RMA)-normalized expression levels of selected genes relevant to osteogenesis (Osteo), hypertrophic cartilage (Hypertrophy), chondrogenesis (Chondro), inhibition of Wnt/β-catenin signaling (Wnt/β-catenin inhibition) and activation of Wnt/β-catenin signaling (Wnt/β-catenin activation) in samples of human primary chondrocytes, BMMSCs and osteoblasts. (F) Relative changes of mean expression levels of selected gene sets and the p value in human primary chondrocytes compared to MSCs and chondrocytes compared to osteoblasts. Genes with significant upregulation (p < 0.05, fold-change>1) are colored in red, and with significant downregulation (p < 0.05, fold-change<−1) are colored in green. (G) Upstream analysis for differential gene expression of human primary chondrocytes compared to MSCs (Chondro versus MSC, top panel) and compared to osteoblasts (Chondro versus Ostb, bottom panel) generated by Ingenuity Pathway Analysis (IPA). Wnt-, TGFβ- and adherens junction-related factors with significance (p < 0.05) are presented.

To assess the physiological relevance of our findings, we performed bioinformatics analyses using human primary BM-MSC, chondrocyte, and osteoblast transcriptome data from public database (GSE108186 for human primary BM-MSCs; GSE68038 for human primary chondrocytes; and GSE121892 for human primary osteoblasts). Initial analyses using principal component analysis (PCA) demonstrated that chondrocytes, osteoblasts, and MSCs are three highly distinct populations (Figure 6A). We then evaluated the transcriptomic changes of MSC lineage specification toward chondrogenic and osteogenic lineages by comparing mRNA expression profiles of chondrocytes and osteoblasts to MSCs (Chondro versus MSC and Ostb versus MSC, respectively). To uncover genes and pathways relevant to cartilage maintenance but not hypertrophy and ossification, we compared the transcriptomic profiles of chondrocytes to osteoblasts (Chondro versus Ostb) (Figure 6B). GSEA based on major developmental pathways^28^ showed that both Wnt and TGFβ family signaling are negatively enriched in Chondro versus MSC (Figure 6C, left panel) and Chondro versus Ostb (Figure 6C, right panel); in contrast, in Ostb versus MSC, Wnt signaling is positively enriched and TGFβ family signaling is non-significantly enriched (Figure 6C, middle panel). These findings implicated that the strategy of Wnt antagonism with removal of TGFβ for MSC chondrogenesis is physiologically relevant and less likely to result in hypertrophy/ossification. In addition, enrichment plots of Wnt and TGFβ family signaling pathways in Chondro versus MSC showed that both pathways are significantly enriched (p<0.05) with normalized enrichment score (NES) of −1.54 and −1.61, respectively (Figure 6D, left two panels). Similarly, in Chondro versus Ostb, Wnt and TGFβ family signaling were also significantly enriched with NES of −1.54 and −1.61, respectively, (Figure 6D, right two panels). We then analyzed by cell type the mRNA expression levels of specific genes in the canonical Wnt/β-catenin pathway, during chondrogenesis, as well as during osteogenesis for further validation of the GSEA results (Figure 6E). To better assess the directionality of expression for each gene, we compared the changes of Robust Multi-array Average (RMA) levels of specific genes in human chondrocytes to that of MSCs (Chondro versus MSC) and osteoblasts (Chondro versus Ostb) (Figure 6F). The results showed that the osteogenic/hypertrophy genes *RUNX2*, *COL1A1* and *ALPL* were significantly downregulated in primary chondrocytes compared to MSCs, and *RUNX2*, *ALPL*, *SPP1*, *COL1A1* and *COL10A1* were downregulated in chondrocytes compared to osteoblasts. In contrast, chondrogenic genes including *SOX5*, *SOX6*, *COL2A1* and *ACAN* were more highly expressed in chondrocytes than MSCs or osteoblasts, with the surprising exception of *SOX9* which was more highly expressed in MSCs and osteoblasts than in chondrocytes. *GSK3B*, an inhibitory gene of the Wnt/β-catenin pathways, were more highly expressed in primary chondrocytes, whereas Wnt/β-catenin downstream genes *AXIN1*, *AXIN2* were less expressed in chondrocytes compared to MSCs or osteoblasts.

Using Upstream Regulator Analysis in Ingenuity Pathway Analysis (IPA) which can provide a more causal relationship between transcription factors/master regulators and downstream pathways,^29^ we found that TGFβ1, Smad2/3, WNT3A, TCF4, BMP2, and TGFBR2 were all predicted to be downregulated transcription factors/master regulators in human primary chondrocytes compared to MSCs. Furthermore, TGFβ1, CTNNB1, and WNT3A were predicted to be downregulated regulators in human primary chondrocytes compared to osteoblasts. Specifically, α-catenin—a core components of the AJ—was predicted as an upregulated regulator in chondrocytes compared to both MSCs and osteoblasts (Figure 6G). All these bioinformatic results were in line with our data demonstrating participation of AJs in WNT/β-catenin antagonism-induced chondrogenesis (Figure 5). Additional validation using another recent published dataset of human primary chondrocyte transcriptomes from various developmental stages^30^ also demonstrated that both TGFβ and Wnt signaling pathways are negatively enriched in adult articular chondrocytes compared to limb bud pre-chondrocytes (Figure S8). Overall, these results underscore that the Wnt/β-catenin pathway as well as the TGFβ pathway is downregulated in primary human chondrocytes compared to osteoblasts and MSCs.

## Discussion

MSC therapy likely offers the best possibility of a curative treatment for cartilage and joint diseases, which currently lacks such significant disease-modifying treatments. However, the relatively more difficult 3D pellet differentiation protocol and the need for using a protein-based growth factor, TGFβ, continue to be obstacles for robust clinical applications. TGFβ is a cytokine with complex functions, and has pleomorphic roles in specification of multiple mesenchymal lineages, not only for chondrogenesis but also osteogenesis.^10^^,^^19^^,^^21^ In many transgenic mouse studies, Wnt inhibition has been clearly shown to promote cartilage development^13^^,^^31^ whereas Wnt activation induces osteogenesis.^12^^,^^32^ Although some early *in vitro* stem cell differentiation studies did not consistently find Wnt inhibition to promote chondrogenesis, these reports utilized biologically derived Wnt inhibitory ligands which are known to have off-target effects as well as potency issues.^33^^,^^34^ In addition, some studies utilized non-MSCs which may not be the appropriate system to investigate chondrogenic lineage specification through Wnt/β-catenin.^33^ Using highly potent small molecule agonists and antagonists of the Wnt/β-catenin pathway, we demonstrated that Wnt/β-catenin antagonism efficiently induce *in vitro* chondrogenesis in human MSCs from multiple sources, and *in vivo* using murine BM-MSCs. Moreover, in comparison with TGFβ3, the most potent member of the family for induction of chondrogenesis, Wnt/β-catenin antagonism more rapidly induced chondrogenesis without inducing other non-chondrogenic, off-target, lineages. Our findings are further supported by transcriptome analysis of primary human MSCs, chondrocytes, and osteoblasts, which demonstrated that the Wnt/β-catenin and TGFβ family signaling pathways were downregulated in chondrocytes relative to the other 2 cell types (Figures 6C–6G). Our findings therefore strongly implicated that Wnt/β-catenin antagonism using potent small molecules can efficiently and more precisely promotes MSC chondrogenesis than TGFβ. Our report of replacing TGFβ agonism with Wnt antagonism increases induction efficiency and allows for the use of more stable and less costly small molecules rather than protein-based factors, and limits off-target lineage commitment which could possibly better preserve the chondrogenic phenotype. Because cost and maintaining lineage in the transplanted cell continue to be major obstacles in cartilage cell therapy,^35^ our findings here could have important translational implications.

We found that the efficient chondrogenic commitment mediated by Wnt/β-catenin antagonism involved strong upregulation of N-cadherin expression and N-cadherin/β-catenin interaction at the AJ which enhanced pellet condensation (Figure 5), a critical step in chondrogenesis and cartilage formation.^36^ Because initiation of chondrogenic induction of MSCs requires better cell-cell interaction rather than cell-matrix adhesion, non-adhesive 3D pellet culture or micromass culture are considered as standard methods to induce MSC chondrogenic differentiation. Previous reports showed that neutralization of N-cadherin results in the inability of mesenchymal cells to condense and therefore inhibits subsequent chondrogenesis^37^; conversely, addition of N-cadherin biomimetic peptides can enhance neocartilage formation by human MSCs.^38^ These findings demonstrated the crucial role of N-cadherin-mediated cell condensation in promoting chondrogenic differentiation. We found that expression of N-cadherin, and interactions between N-cadherin and β-catenin were significantly upregulated with Wnt inhibition during MSC chondrogenic differentiation (Figure 5), implying that Wnt/β-catenin antagonism can increase N-cadherin levels and cell condensation, both critical processes for chondrogenesis. Moreover, disruption of actin polymerization—also a critical process during *in vivo* condensation^39^—by CytoD was partially rescued with Wnt/β-catenin antagonism (Figures 5F and 5G), demonstrating the key role of this pathway on multiple aspects of chondrogenesis. Even more striking, using Upstream Regulator Analysis which predict involvement of transcription factors/master regulators, α-catenin was predicted as an upregulated factor, whereas TGFβ as well as Wnt/β-catenin members were predicted to be downregulated in primary chondrocytes compared to MSCs as well as osteoblasts (Figure 6G). Our findings therefore strongly implicate the critical role of β-catenin as a structural protein as well as further support the importance of N-cadherin in the AJ and the cytoskeleton-mediated condensation during MSC chondrogenesis.

Our study revealed the many off-target lineages specification by TGFβ during MSC chondrogenesis. One of the most important molecules/pathways in developmental biology, TGFβ is known to be involved in specification of multiple mesodermal lineages, as well as mediate pathological fibrotic processes. In the development of the skeletal system, TGFβ not only induces chondrogenic differentiation and modulates the process of hypertrophy^40^ but also osteogenic differentiation as well.^19^ In addition, TGFβ promotes differentiation of MSCs into smooth muscle cells^9^; this was clearly reflected in our transcriptomic analysis which showed that signaling by TGFβ family was highly enriched in SMCs compared to MSCs (Figures 2A and 2B). Of interest, the evidence for involvement of TGFβ signaling in chondrogenesis appears to be largely *in vitro*, because no defect in cartilage formation seen in either TGFβ1-or TGFβ3-deficient mice.^8^ Despite the well documented evidence on the pleomorphic effects of TGFβ on multiple mesenchymal lineages, no study has concurrently evaluated commitment into these lineages. We found that even under chondrogenic 3D pellet culture conditions, TGFβ increased the expression of both the smooth muscle-lineage marker *α*SMA and the osteogenic/hypertrophic marker *RUNX2* (Figure 2). Such significant off-target lineage specifications may contribute to a lower chondrogenic differentiation efficiency, and be responsible for ossification and/or fibrosis observed in transplanted chondrocytes derived from TGFβ-differentiated MSCs.^41^^,^^42^ Although two recent studies reported that removal of TGFβ and/or applying Wnt antagonist in the late stage of MSC chondrogenic differentiation reduced hypertrophic cartilage formation,^43^^,^^44^ we found that addition of TGFβ rapidly upregulated canonical Wnt/β-catenin downstream genes *AXIN2* and *TCF7* in addition to *RUNX2*—the upstream transcription factor controlling the hypertrophic phenotype^45^^,^^46^—as early as Day 3 of the differentiation period (Figures 4C and 4D), which is in line with previous reports that TGFβ is a weak agonist for Wnt/β-catenin signaling.^47^ Moreover, the use of MSCs rather than pluripotent stem cells may lead to less non-chondrogenic specification and a more uniformed outcome.^44^ Our findings with previous transgenic mice data collectively support that Wnt/β-catenin antagonism rather than TGFβ agonism may be the most critical and appropriate pathway to efficiently and specifically induce MSC chondrogenic lineage commitment.

The master transcription factor for chondrogenesis is SOX9,^48^ however, we did not find further upregulation of this transcription factor with Wnt inhibition during MSC chondrogenic induction using 3D pellet culture (Figure 1C), unlike the significant upregulation of more downstream chondrogenic genes *COL2A1* and *ACAN* (Figures 1D and 1E). Our *in vitro* data was surprisingly corroborated in transcriptome analysis of human samples, in which *SOX9* was downregulated in chondrocytes compared to osteoblasts and undifferentiated MSCs (Figures 6E and 6F). Similar to our data, a discrepant trend in expression levels between *SOX9* and mature chondrogenic markers including *COL2A1* and *ACAN* has been reported previously.^49^^,^^50^^,^^51^ These findings collectively strongly suggest that high levels of Sox9 expression are a necessary event in development and early stages of chondrogenesis, but less evident at later stages including *in vitro* differentiation in somatic progenitors/stem cells such as MSCs.

**Figure 7:**
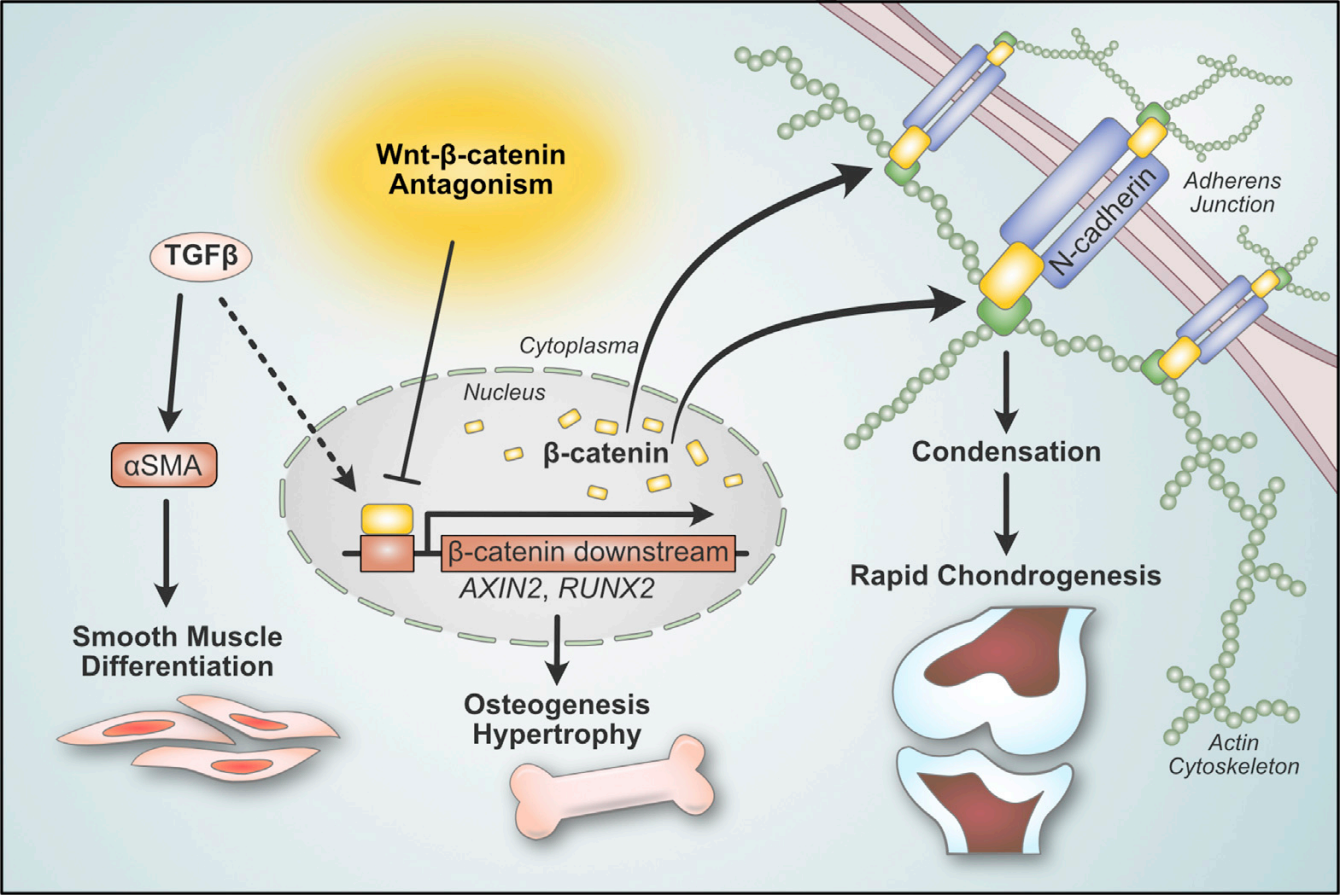
Wnt/β-catenin antagonism induce robust and specific MSC chondrogenesis Wnt/β-catenin antagonism induces rapid chondrogenic differentiation by restricting osteogenic lineage commitment and enhancing pellet condensation through increasing N-cadherin expression and N-cadherin/β-catenin interactions at the AJ. The removal of TGFβ further avoids off-target specification toward osteogenesis/hypertrophy, fibrosis, and smooth muscle differentiation.

In summary, using multiple sources of human MSCs including ESC-MSCs and iPSC-MSCs we found that replacement of TGFβ agonism with Wnt/β-catenin antagonism resulted in robust and specific *in vitro* and *in vivo* MSC chondrogenesis by eliminating off-target lineage specification into osteogenesis/hypertrophic cartilage and smooth muscle. Wnt/β-catenin antagonism also more efficiently induced MSC chondrogenesis by increasing N-cadherin levels as well as N-cadherin-β-catenin interactions at the AJ to enhance condensation (Figure 7). These findings are also corroborated by bioinformatic analysis of human primary MSC, chondrocyte, and osteoblast transcriptomes, in which downregulation of both Wnt/β-catenin and TGFβ pathways, with upregulation of α-catenin-related processes was seen in primary chondrocytes compared to MSCs and osteoblasts. Our study underscores the importance of structural modification in MSC chondrogenesis, as well as having a thorough understanding of key developmental pathways in lineage specification. The capacity to use small molecules rather than a protein growth factor for stem cell differentiation is also more cost-effective, and therefore highly relevant for translational application.

### Limitations of the study

In this study, we found that Wnt antagonism alone can induce MSC chondrogenic differentiation through increasing AJ interactions and condensation, whereas restricting off-target lineage commitment which can occur with the classical differentiation protocol using TGFβ. But the detailed molecular mechanisms involved are still unclear and should be clarified in future studies. Moreover, the aim of our animal experimental results is to provide *in vivo* proof-of-concept. The therapeutic efficacy of Wnt antagonism for MSC chondrogenesis should be further investigated in disease models with cartilage defects.

## STAR★Methods

### Key resources table

**Table:** 

REAGENT or RESOURCE	SOURCE	IDENTIFIER
**Antibodies**		

Anti-Actin, α-Smooth Muscle	Sigma-Aldrich	Cat#F3777; RRID:AB_476977
Purified Mouse Anti-β-Catenin	BD Biosciences	Cat#610154; RRID:AB_397555
Anti-N cadherin antibody	Abcam	Cat#ab18203
Anti-Collagen II antibody	Abcam	Cat#ab34712; RRID:AB_731688
Anti-Aggrecan antibody	Abcam	Cat#ab3778; RRID:AB_304071
N-cadherin blocking antibody	Sigma-Aldrich	Cat#C3865; RRID:AB_262097

**Chemicals, peptides, and recombinant proteins**		

Recombinant Human TGF-beta 1 Protein	R&D	Cat#240-B
Recombinant Human TGF-beta 3 Protein	R&D	Cat#243-B3
XAV939	Sigma-Aldrich	Cat#X3004
CHIR99021	Sigma-Aldrich	Cat#SML1046
iCRT-3	Sigma-Aldrich	Cat#SML0211
LiCl	Sigma-Aldrich	Cat#L9650
Alcian blue 8GX	Sigma-Aldrich	Cat#A3157
Cytochalasin D	Sigma-Aldrich	Cat#C8273

**Critical commercial assays**		

Duolink *In Situ* PLA kit: Duolink *In Situ* Detection Reagents Red	Sigma-Aldrich	Cat#Duo92008
Duolink *In Situ* PLA kit: Duolink *In Situ* PLA Probe Anti-Rabbit PLUS	Sigma-Aldrich	Cat#Duo92002
Duolink *In Situ* PLA kit: Duolink *In Situ* PLA Probe Anti-Mouse MINUS	Sigma-Aldrich	Cat#Duo92004

**Deposited data**		

Raw and Analysis data	This paper	GEO: GSE211559
Raw and Analysis data of human primary BM-MSCs	N/A	GEO: GSE128949
Raw and Analysis data of human primary SMCs	N/A	GEO: GSE109859
Raw and Analysis data of human primary BM-MSCs	Yoshida et al. 2019^52^	GEO: GSE108186
Raw and Analysis data of human primary chondrocytes	Stradner et al. 2016^53^	GEO: GSE68038
Raw and Analysis data of human primary osteoblasts	De-Ugarte et al. 2018^54^	GEO: GSE121892

**Experimental models: Cell lines**		

Human bone marrow MSC	PromoCell	Cat#C-12974
Human ESC-derived MSC	Yen et al. 2009^5^	N/A
Human iPSC-derived MSC	Wang et al. 2018^7^	N/A

**Experimental models: Organisms/strains**		

Mouse C57BL/6JNarl	National Laboratory Animal Center of Taiwan	RMRC11005

**Software and algorithms**		

Image J	National Institutes of Health, USA	https://imagej.nih.gov/ij/
Prism	GraphPad	https://www.graphpad.com/scientific-software/prism/
GSEA	Subramanian et al., 2005^55^	https://www.gsea-msigdb.org/gsea/index.jsp
Partek Genomics Suites	Partek	https://www.partek.com/partek-genomics-suite/

### Resource availability

#### Lead contact

Further information and requests for resources and reagents should be directed to and will be fulfilled by the lead contact, B. Linju Yen (blyen@nhri.org.tw).

#### Materials availability

This study did not generate new unique reagents.

### Experimental model and subject details

#### Cell lines

Human iPSC-MSCs were derived from iPSCs generated from fetal endothelial cells through lentiviral transduction of *OCT-4* and *SOX-2*,^56^ and human ESC-MSCs were derived from H1 (Wisconsin Alumni Research Foundation, Madison, WI, USA) with medium consisted of DMEM-low glucose, 1% penicillin/streptomycin and 10% fetal bovine serum.^5^^,^^7^^,^^57^ BM-MSCs were obtained from commercial sources (Promocell, Heidelberg, Germany). All MSCs were cultured and expanded in low-glucose Dulbecco’s Modified Eagle’s medium (DMEM) (Gibco-Thermo Fisher Scientific, MA, USA), with 10% FBS (Hyclone-Thermo Fisher Scientific) and 100 U/mL penicillin, 100 g/mL streptomycin, and 2 mM L-glutamine (all from Gibco-Thermo Fisher Scientific).^3^^,^^58^

#### Mouse model

##### *In vivo* chondrogenesis

All animal work was performed according to protocols approved by the Institutional Animal Care and Use Committee. *In vivo* ectopic chondrogenesis was performed as previously reported with modifications.^59^ Mouse BM-MSCs were cultured as 3D pellets in ChBM alone or with addition of TGFβ3, CHIR, or XAV for 3 days, then subcutaneously transplanted to dorsal skin of wild-type C57BL/6J mice, with local injections of modulators every 3 days till the pellet sample was harvested at Day 20. Collected samples were then frozen in optimal cutting temperature (OCT) compound and sliced to 5 mm in thickness for histological staining.

### Method details

#### *In vitro* chondrogenic differentiation

*In vitro* chondrogenic differentiation using 3D pellet culture was performed as described previously,^7^^,^^60^^,^^61^ 2 × 10^5^ trypsinized MSCs were centrifuged 450 × g for 10 minutes to form a pellet in expansion medium. After 16 hours, the medium was changed to chondrogenic basal medium (ChBM) consisting of low-glucose DMEM supplemented with 100 units/mL penicillin, 100 μg/mL streptomycin (all from Gibco-Thermo Fisher Scientific), 1% Insulin-transferrin-sodium selenite media supplement (Sigma-Aldrich, St Louis, MO, USA), 2 mM L-glutamine (Gibco-Thermo Fisher Scientific), 10 μM L-Ascorbic acid 2-phosphate (Sigma-Aldrich), 100 nM dexamethasone (Sigma-Aldrich). Addition of TGFβ1 (10 ng/mL; R&D Systems, Minneapolis, MN, USA), TGFβ3 (10 ng/mL; R&D Systems), XAV939 (XAV; 10 μM; Sigma-Aldrich) or CHIR99021 (CHIR; 10 μM; Sigma-Aldrich) to ChBM was performed as indicated. Cell pellets were harvested at the indicated day for analysis. Micromass culture-based chondrogenic differentiation was performed by applying 2 × 10^7^ cells/mL of MSCs suspended in expansion medium and 20-μL drops spotted in the center of each well of a 24-well culture plate.^22^^,^^62^ After adhesion for 1.5 hour in a humidified incubator at 37°C with 5% CO_2_, the medium was changed to ChBM with indicated regulators for chondrogenic induction.

#### Alcian Blue staining and quantification

Samples were washed with PBS and fixed with 4% paraformaldehyde, followed by pH 1.0 1% Alcian blue 8GX (Sigma-Aldrich) staining at room temperature overnight to detect sulfated proteoglycan matrix.^63^ Quantification of Alcian blue staining extracted with 6M Guanidine-HCl was measured in 650 nm absorbance.^64^

#### Quantitative real-time PCR

Total RNA was isolated using TRI® reagent (Sigma-Aldrich), and quantified using NanoDrop spectrophotometer (Nyxor Biotech, Paris, France).^65^ Reverse transcription was performed using RevertAid H Minus reverse transcriptase (Thermo Fisher Scientific), and PCR was performed on ABI PRISM 7500 system (Applied Biosystem, Foster City, CA, USA) with SYBR Fast qPCR kit (KAPA Biosystems, Boston, MA, USA). mRNA expression level was calculated by using the ΔΔCt method, and glyceraldehyde 3-phosphate dehydrogenase (*GAPDH*) level were used as housekeeping control. The results are normalized to ChBM to represent effects of indicated modulators. The sequences of primers for each gene are listed in Table S1.

#### Immunofluorescent staining

Immunofluorescent staining was performed as previously reported.^65^ Paraformaldehyde-fixed cell samples were permeabilized with 0.1% Triton X-100 and nonspecific binding was blocked by 5% bovine serum albumin. Primary antibodies against alpha smooth muscle actin (*α*SMA) (Sigma-Aldrich), β-catenin (BD Biosciences, San Jose, CA, USA) or N-cadherin (Abcam, Cambridge, UK) were stained with 1:100 dilution for 24 hours, followed by species specific secondary antibody incubation overnight. Fluorescent signals were acquired by confocal microscopy (Leica TCS SP5 II, Wetzlar, Germany) and analyzed by ImageJ (NIH, Maryland, USA).

#### Proximity ligation assay (PLA)

Micromass cultured iPSC-MSCs were used for PLA and performed according to the manufacturer’s protocol (Sigma-Aldrich). Antibodies against N-cadherin (Abcam) and β-catenin (BD Biosciences) were used for recognition. After ligation and amplification, the PLA signals from each pair of probes in close proximity (<40 nm) were visualized by confocal microscope (Leica TCS SP5 II) and signal counts were analyzed by ImageJ.

#### Gene expression and pathway analysis

Transcriptome data of human primary smooth muscle cells (GSE109859), MSCs (GSE128949 and GSE108186),^52^ chondrocytes (GSE68038),^53^ and osteoblasts (GSE121892)^54^ were obtained from public database. Datasets are merged to perform principal component analysis (PCA) using Partek® Genomics Suite (St. Louis, MO, USA). Ranked Gene Set Enrichment Analysis (GSEA) was performed with software version 4.1.0.^55^ Upstream Analysis were performed using IPA software (Qiagen, https://www.qiagenbioinformatics.com).

### Quantification and statistical analysis

All data represent three replicates or more from separate experiments. Analysis of variance (ANOVA) followed by Tukey’s post-hoc test was performed to evaluate significance for comparisons for multiple groups, with *p*< 0.05 as significant. Analyses were performed using GraphPad Prism software and data are shown as mean ± standard deviation (S.D.).

## Data Availability

• All transcriptomic data generated in this study have been deposited to NCBI GEO database. The accession number is GSE211559. • This paper does not report original code. • Any additional information required to reanalyze the data reported in this paper is available from the lead contact upon request.
