# Supplementary material for: Wnt antagonism without TGFβ induces rapid MSC chondrogenesis via increasing AJ interactions and restricting lineage commitment
Source: iScience. 2022 Dec 2;26(1):105713. doi: 10.1016/j.isci.2022.105713 (PMC9792887; doi:10.1016/j.isci.2022.105713)
Supplement: Document S1. Figures S1–S8 and Table S1 [file mmc1.pdf]

iScience, Volume *26*

Supplemental information

Wnt antagonism without TGFβ induces rapid MSC chondrogenesis via increasing AJ interactions and restricting lineage commitment

Chen-Chan Hsieh, B. Linju Yen, Chia-Chi Chang, Pei-Ju Hsu, Yu-Wei Lee, Men-Luh Yen, Shaw-Fang Yet, and Linyi Chen

Table S1. Primer sets for quantitative real-time PCR, Related to Method Details.

Gene Name	Forward	Reverse
SOX9	5’-TTTCCAAGACACAAACATGA-3’	5’-AAAGTCCAGTTTCTCGTTGA-3’
COL2A1	5’-GGCAATAGCAGGTTCACGTACA-3’	5’-CGATAACAGTCTTGCCCCACTT-3’
ACAN	5’-TCGAGGACAGCGAGGCC-3’	5’-TCGAGGGTGTAGCGTGTAGAGA-3’
RUNX2	5’- GGTTAATCTCCGCAGGTCACT -3’	5’-CACTGTGCTGAAGAGGCTGTT-3’
AXIN2	5’-AGTGTGAGGTCCACGGAAAC-3’	5’-CTGGTGCAAAGACATAGCCA-3’
TCF7	5’-CTGGAGAAGCTCAAAGGCC-3’	5’-TTGGGTGGTAAGTCAGTGTCC-3’
GAPDH	5’-CCACCCATGGCAAATTCCATGGCA-3’	5’-TCTAGACGGCAGGTCAGGTCCACC-3’

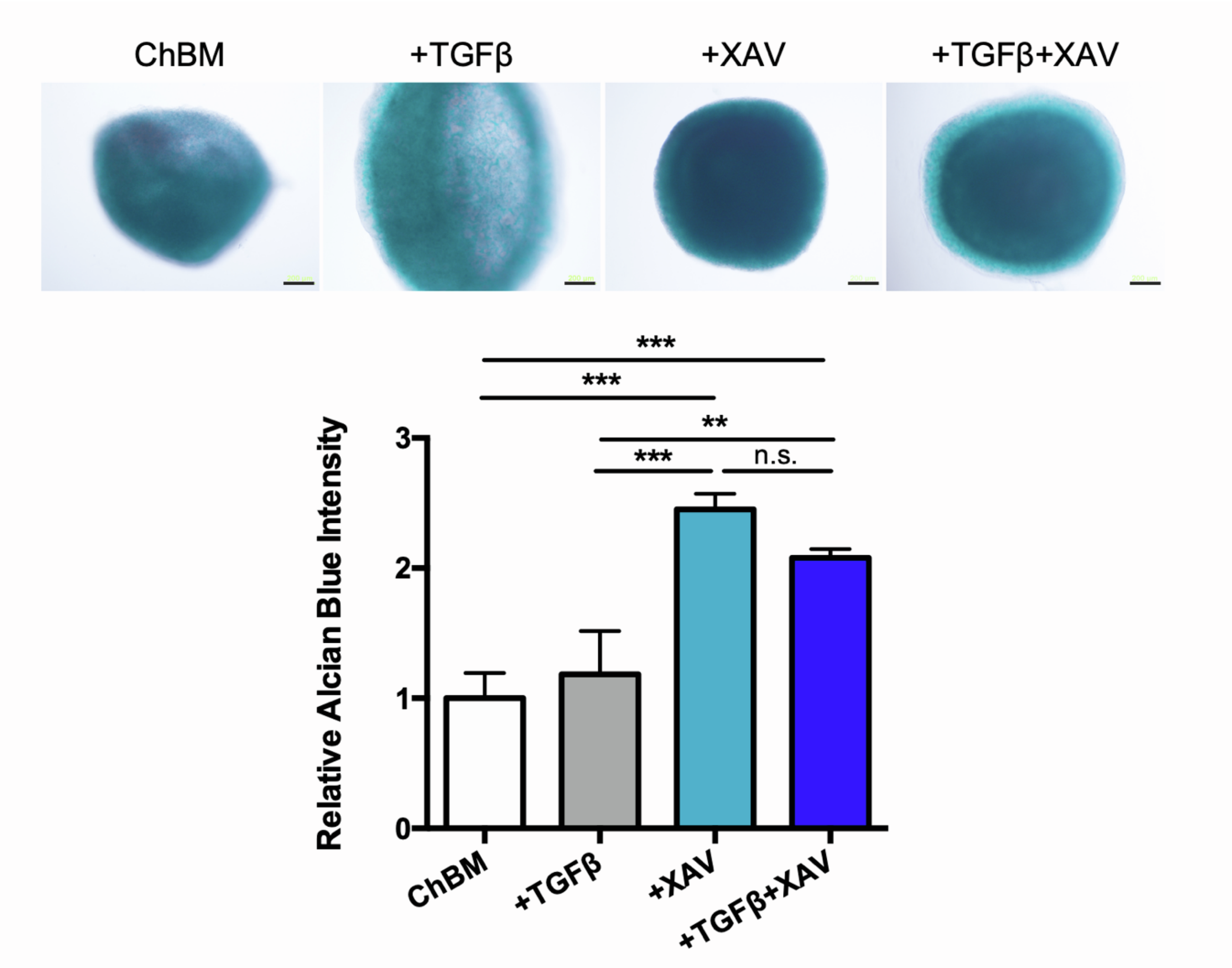

Figure S1. Wnt/β-catenin antagonism alone significantly increases MSC chondrogenesis at an earlier time point which is not improved with TGFβ3 supplementation, Related to Figure 3. Alcian Blue staining (top panel) and absorbance quantification (bottom panel) of pellet-cultured iPSC-MSCs treated with the indicated modulators at Day 10. Scale bar, 500 µm. Data are represented as mean +/- SD. One-way ANOVA: **, $p < 0.01$, ***, $p < 0.001$, n.s., not significant.

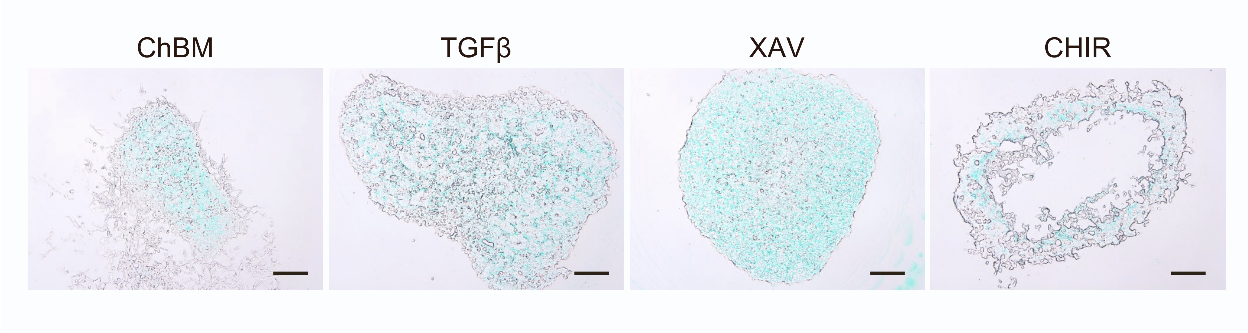

Figure S2. Wnt/β-catenin antagonism alone induced more rapid MSC chondrogenesis than TGFβ, Related to Figure 3. Alcian Blue staining of sliced pellet (10-μm thickness) of iPSC-MSCs treated with the indicated modulators (10 ng/mL TGFβ3, 10 μM CHIR, or 10 μM XAV) at Day 10. Scale bar, 100 μm.

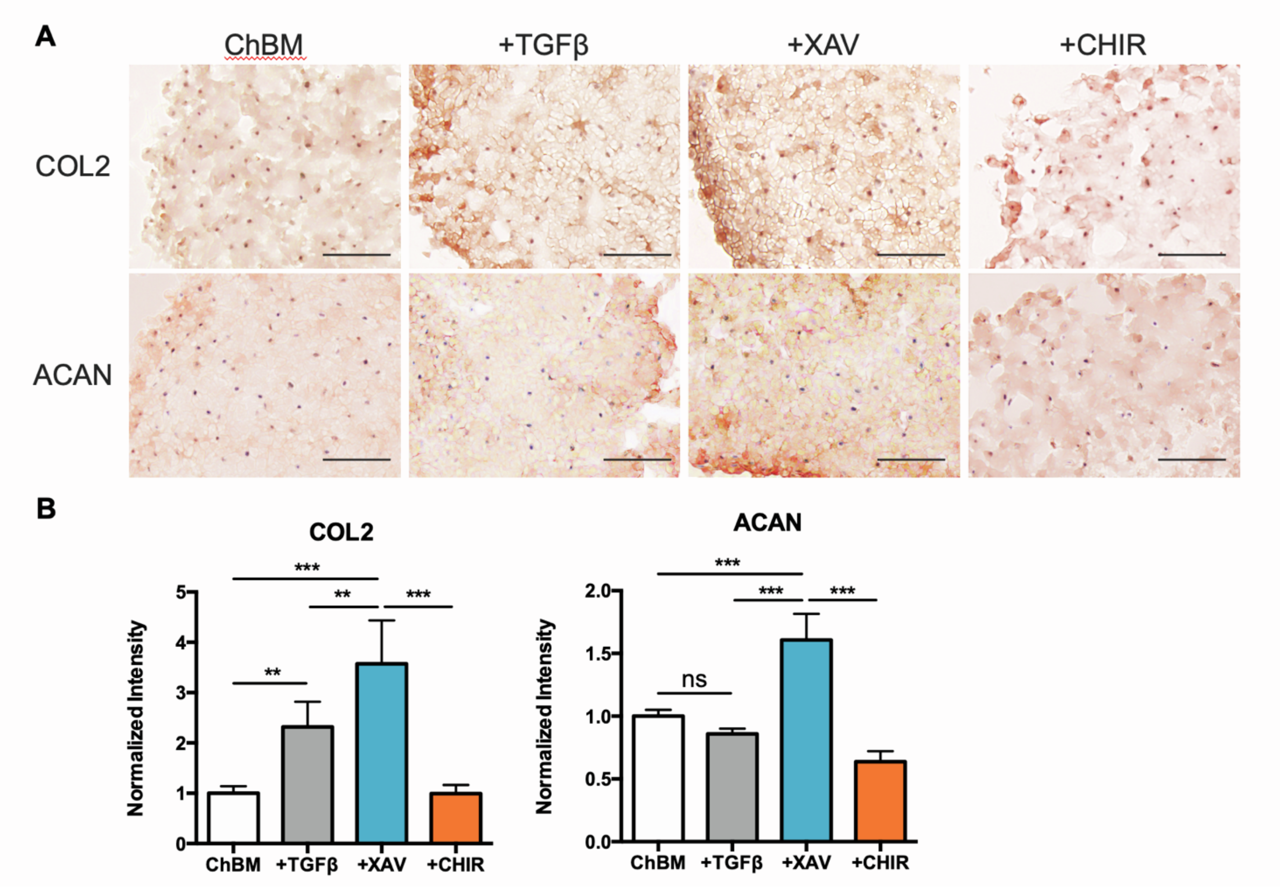

Figure S3. Wnt/β-catenin antagonism alone induced more rapid MSC chondrogenesis than TGFβ, Related to Figure 3. (A) Immunohistochemical staining for type II collagen (COL2) and aggrecan (ACAN) of sliced pellet (10-μm thickness) of iPSC-MSCs treated with the indicated modulators (10 ng/mL TGFβ3, 10 μM XAV, or 10 μM CHIR) at Day 10. Scale bar, 100 μm. (B) Relative signal intensity as measured by Image J. Data are represented as mean +/- SD. One-way ANOVA: **, $p < 0.01$, ***, $p < 0.001$, ns, not significant.

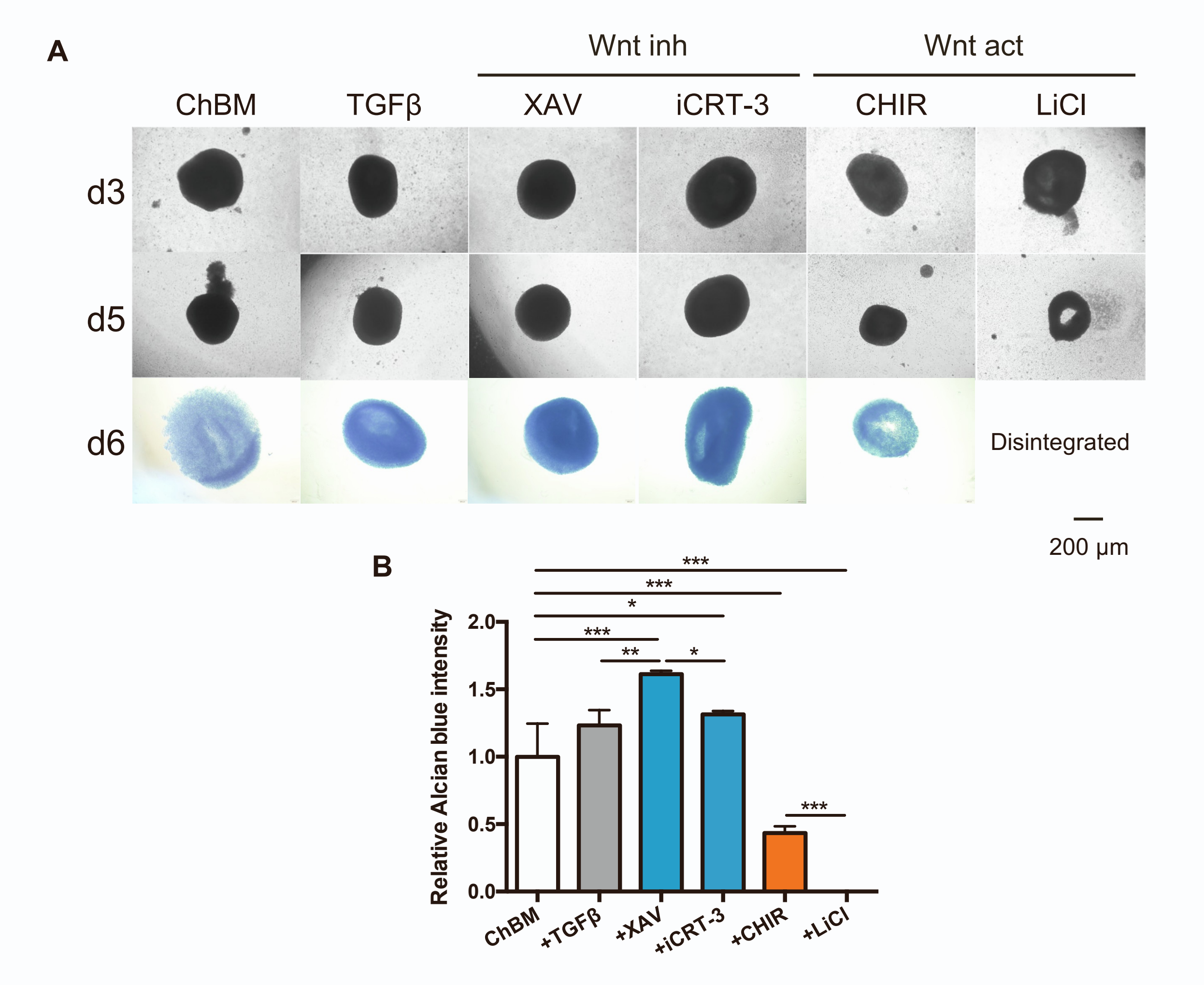

Figure S4. Wnt/β-catenin antagonists XAV and iCRT-3 enhance hMSC chondrogenesis while agonists CHIR and lithium chloride (LiCl) suppressed chondrogenesis, Related to Figure 3.

(A) Phase contrast images at Day 3 (d3), Day 5 (d5), and Day 6 after Alcian Blue staining (d6) as well as (B) Absorbance quantification of iPSC-MSC pellets treated with the indicated modulators (10 ng/mL TGFβ3, 25 nM iCRT-3, 10 µM XAV, 10 µM CHIR, or 30 mM LiCl) at Day 10. Data are represented as mean +/- SD. One-way ANOVA: *, $p < 0.05$, **, $p < 0.01$, ***, $p < 0.001$.

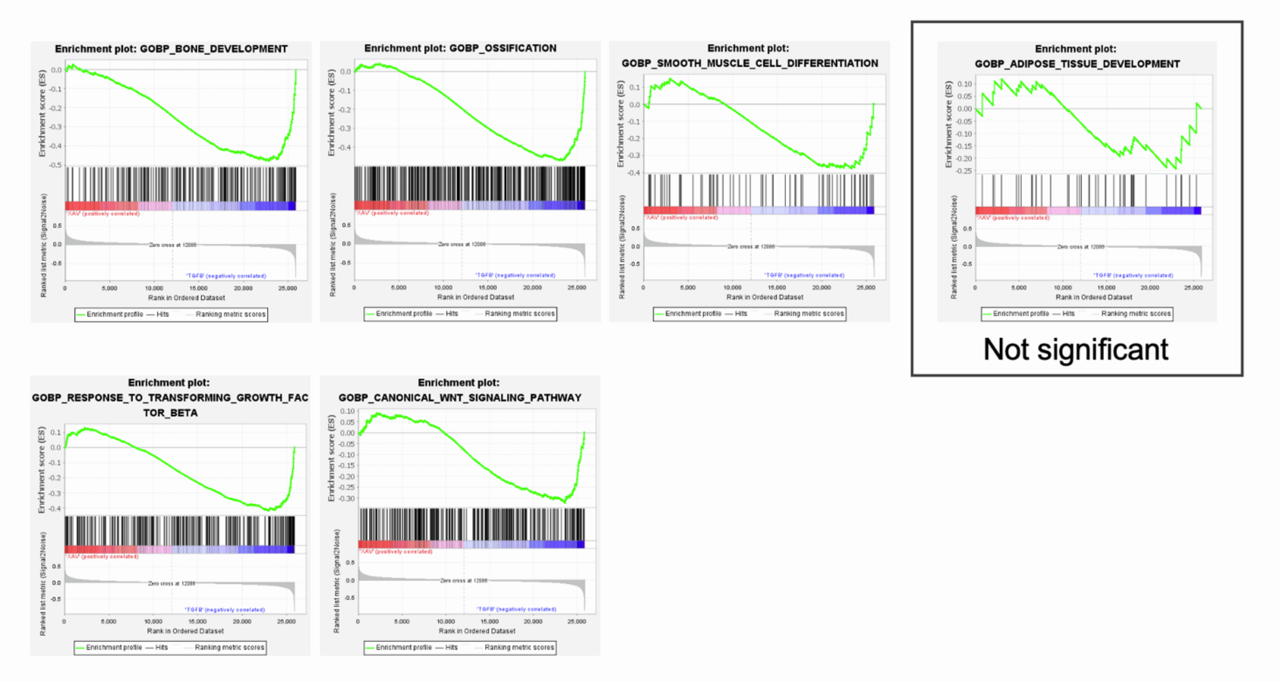

Figure S5. Gene sets of Bone development, Ossification, and Smooth muscle cell differentiation are negatively enriched, and that of Adipose tissue development is not significantly enriched in hMSCs with XAV-based chondrogenic induction compared to TGFβ treatment, Related to Figure 3. GSEA enrichment plot of Bone development, Ossification, Smooth muscle cell differentiation, Response to TGFβ, and Canonical Wnt signaling pathway in transcriptomes of hMSCs (2 iPSC-MSCs and 1 BM-MSC) after three days of XAV-based chondrogenic induction compared to TGFβ treatment.

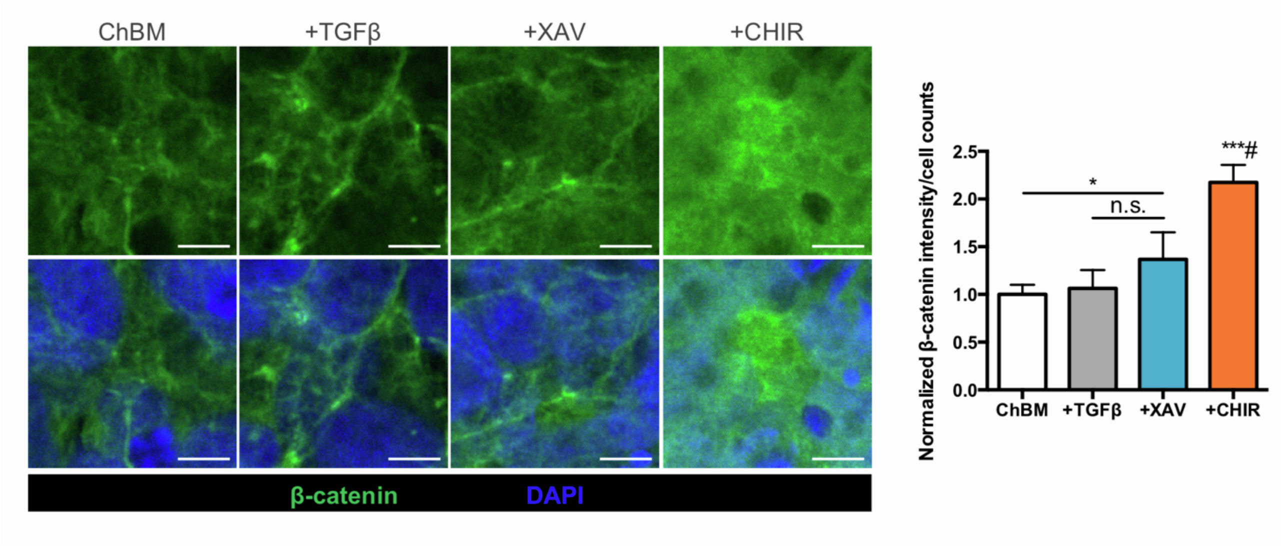

Figure S6. β-catenin expression levels after Wnt/β-catenin or TGFβ modulation in iPSC-MSCs undergoing chondrogenesis, Related to Figure 5. Confocal immunofluorescence microscopy and fluorescence quantification of β-catenin in micromass-cultured iPSC-MSCs with the indicated modulators in chondrogenic induction medium for 1 day. Nuclei are labeled by DAPI. Scale bar, 5 μm. ChBM, chondrogenic basal medium without TGFβ. XAV, Wnt/β-catenin antagonist XAV939. CHIR, Wnt/β-catenin agonist CHIR99021. Data are represented as mean +/- SD. One-way ANOVA: *, $p < 0.05$, **, $p < 0.01$, ***, $p < 0.001$.

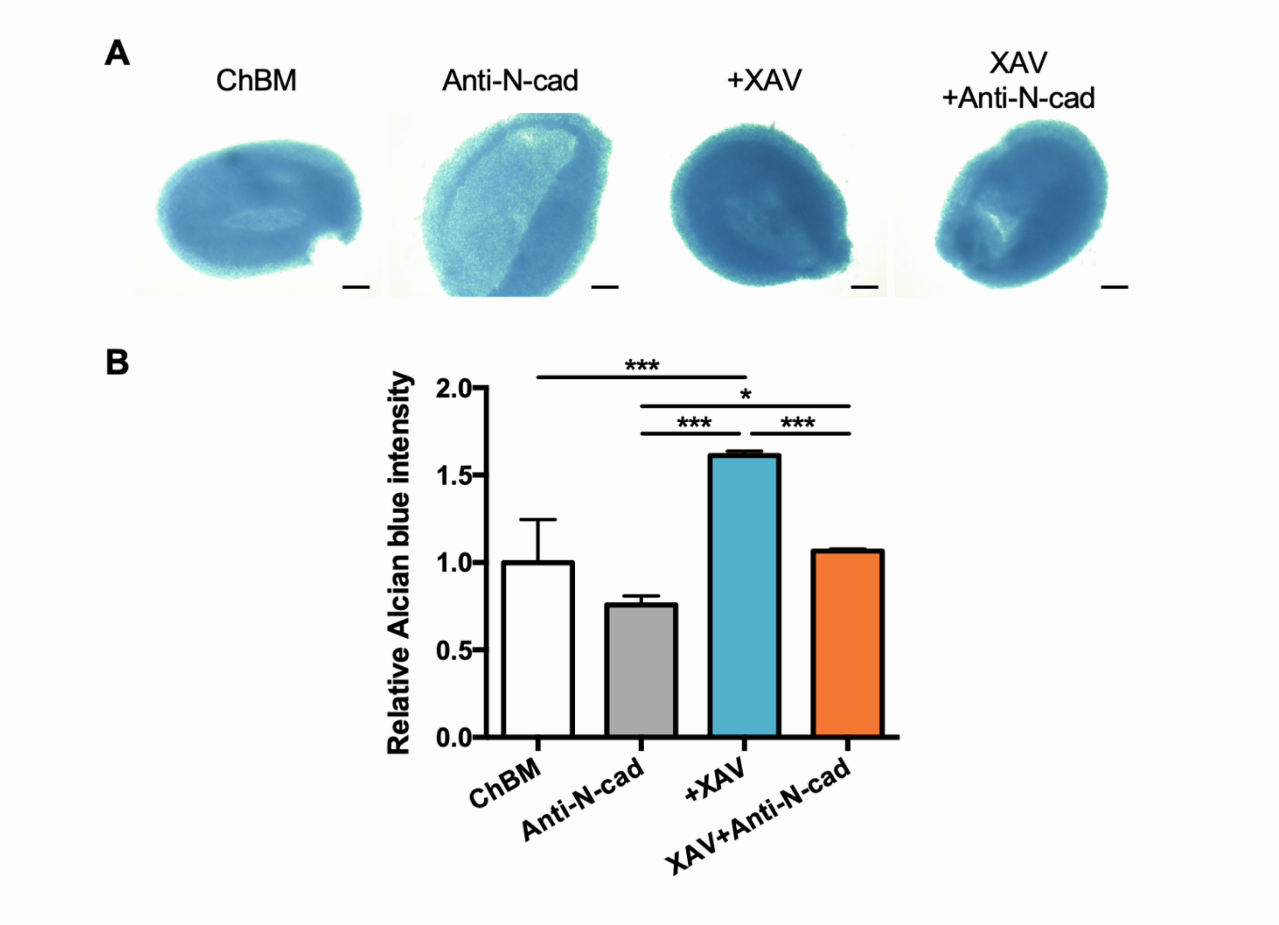

Figure S7. Blocking of N-cadherin abrogates Wnt antagonism-induced chondrogenesis, Related to Figure 5. (A) Alcian Blue staining and (B) the absorbance quantification of iPSC-MSC pellets treated with the indicated modulators (1:50 anti-N-cadherin antibody (Anti-N-cad) and/or 10 µM XAV) at Day 6. Data are represented as mean +/- SD. One-way ANOVA: *, $p < 0.05$, ***, $p < 0.001$.

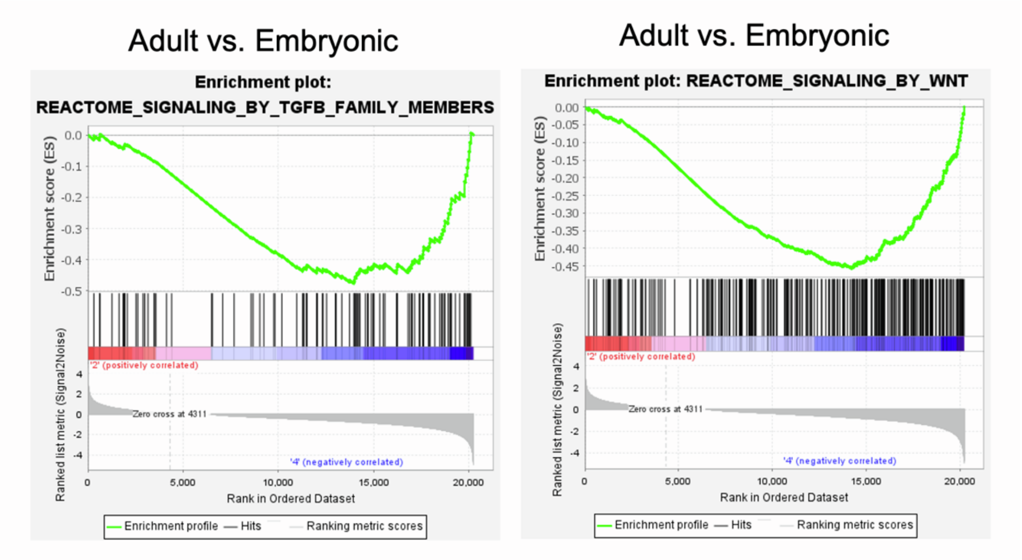

Figure S8. Both TGFβ and Wnt signaling pathways are negatively enriched in adult articular chondrocytes compared to limb bud pre-chondrocytes as well as fetal chondrocytes, Related to Figure 6. GSEA enrichment plot of signaling by TGFβ family members (stable Identifier: R-HSA-9006936, left 2 panels) and Wnt (stable Identifier: R-HSA-195721, right 2 panels) in human adult articular chondrocytes (Adult) compared to embryonic limb bud pre-chondrocytes (Embryonic). The raw transcriptomic data was obtained from GSE106292 (Ferguson et al., Nat Comm 2018, doi: 10.1038/s41467-018-05573-y).
